# A Sparsity-Driven Backpropagation-Less Learning Framework Using Populations of Spiking Growth Transform Neurons

**DOI:** 10.3389/fnins.2021.715451

**Published:** 2021-07-28

**Authors:** Ahana Gangopadhyay, Shantanu Chakrabartty

**Affiliations:** Department of Electrical and Systems Engineering, Washington University in St. Louis, St. Louis, MO, United States

**Keywords:** energy-based learning, spiking neural network, neuromorphic machine learning, supervised leaning, domain description, spike rates, sparsity, unsupervised learning

## Abstract

Growth-transform (GT) neurons and their population models allow for independent control over the spiking statistics and the transient population dynamics while optimizing a physically plausible distributed energy functional involving continuous-valued neural variables. In this paper we describe a backpropagation-less learning approach to train a network of spiking GT neurons by enforcing sparsity constraints on the overall network spiking activity. The key features of the model and the proposed learning framework are: (a) spike responses are generated as a result of constraint violation and hence can be viewed as Lagrangian parameters; (b) the optimal parameters for a given task can be learned using neurally relevant local learning rules and in an online manner; (c) the network optimizes itself to encode the solution with as few spikes as possible (sparsity); (d) the network optimizes itself to operate at a solution with the maximum dynamic range and away from saturation; and (e) the framework is flexible enough to incorporate additional structural and connectivity constraints on the network. As a result, the proposed formulation is attractive for designing neuromorphic tinyML systems that are constrained in energy, resources, and network structure. In this paper, we show how the approach could be used for unsupervised and supervised learning such that minimizing a training error is equivalent to minimizing the overall spiking activity across the network. We then build on this framework to implement three different multi-layer spiking network architectures with progressively increasing flexibility in training and consequently, sparsity. We demonstrate the applicability of the proposed algorithm for resource-efficient learning using a publicly available machine olfaction dataset with unique challenges like sensor drift and a wide range of stimulus concentrations. In all of these case studies we show that a GT network trained using the proposed learning approach is able to minimize the network-level spiking activity while producing classification accuracy that are comparable to standard approaches on the same dataset.

## 1. Introduction

As the deployment of miniaturized and battery-powered sensors and devices becomes ubiquitous, computation is increasingly moving from the cloud to the source of data collection. With it, there is a growing demand for specialized algorithms, hardware, and software—collectively termed as tinyML systems—that can perform learning and inference at the edge under energy and resource-constrained environments. Recent efforts at reducing the energy requirements of classical machine learning algorithms include network architecture search (Cai et al., 2018), model compression through energy-aware pruning and quantization (Molchanov et al., 2016; Yang et al., 2017; Oh et al., 2018), model partitioning (Kang et al., 2017), and many more.

Neuromorphic systems, on the other hand, naturally lend themselves to resource-efficient computation, deriving inspiration from tiny brains (insect brains) that not only occupy a small form-factor but also exhibit high energy-efficiency (Chittka and Niven, 2009; Theobald, 2014). Over the last few years, neuromorphic algorithms using event-driven communication on specialized hardware have been claimed to outperform their classical counterparts running on traditional hardware in energy costs by orders of magnitude in bench-marking tests across applications (Stromatias et al., 2015; Marti et al., 2016; Blouw et al., 2019; Tang et al., 2019). However, like traditional ML approaches, these advantages in energy-efficiency were demonstrated only during inference. Implementing spike-based learning and training has proven to be a challenge and in literature one of the following approaches have been reported:

- For a vast majority of energy-based learning models, backpropagation remains the tool of choice for training spiking neural networks. In order to resolve differences due to continuous-valued neural outputs in traditional neural networks and discrete outputs generated by spiking neurons in their neuromorphic counterparts, transfer techniques that map deep neural nets to their spiking counterparts through rate-based conversions are widely used (O'Connor et al., 2013; Diehl et al., 2015; Rueckauer et al., 2016). Other approaches use temporal coding to formulate loss functions that penalize the difference between actual and desired spike-times (Xin and Embrechts, 2001; Bohte et al., 2002; Belatreche et al., 2003; Mostafa, 2017; Zhou et al., 2021), or approximate derivatives of spike signals through various means to calculate error gradients for backpropagation (Lee et al., 2016; Shrestha and Orchard, 2018; Zenke and Ganguli, 2018).
- On the other hand there are neuromorphic algorithms that use local learning rules like the Synaptic Time-Dependent Plasticity (STDP) for learning lower-level feature representations in spiking neural networks. Some of these are unsupervised algorithms that combine the learned features with an additional layer of supervision using separate classifiers or spike counts (Masquelier and Thorpe, 2007; Diehl and Cook, 2015; Kheradpisheh et al., 2016). Yet others adapt weights in specific directions to reproduce desired output patterns or templates in the decision layer, for example, a spike (or high firing rate) in response to a positive pattern and silence (or low firing rate) otherwise. Examples include supervised synaptic learning rules like the tempotron (Gütig and Sompolinsky, 2006) implementing temporal credit assignments according to elicited output responses; and algorithms using teaching signals to drive outputs in the decision layer (Brader et al., 2007; Beyeler et al., 2013).

**Figure 1:**
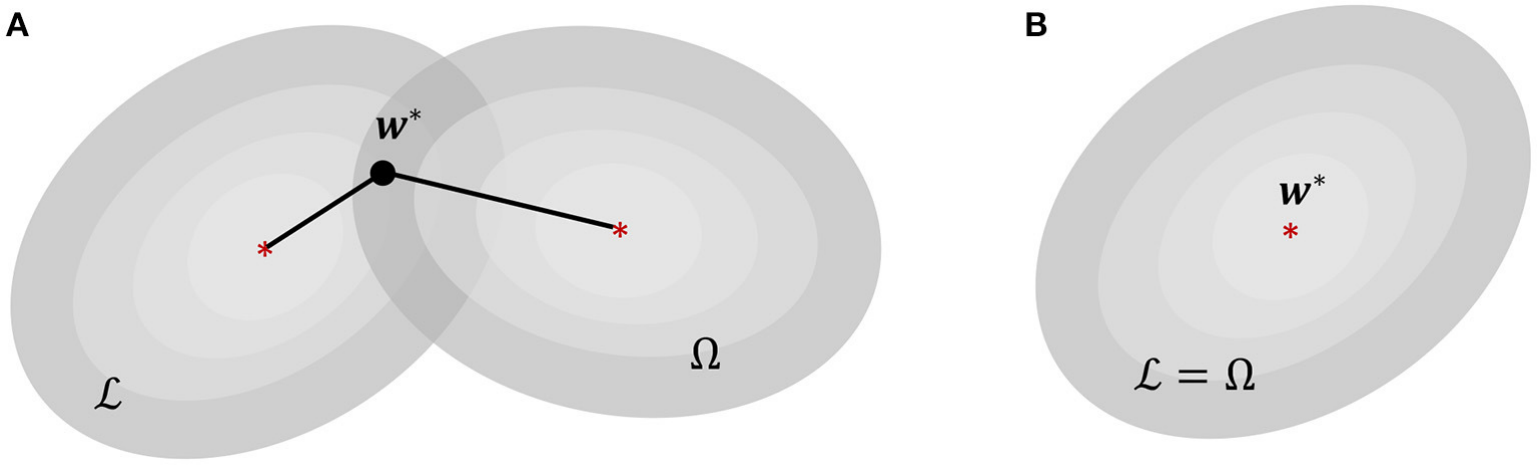
**(A)** Energy-efficiency in energy-based neuromorphic machine learning, where L is the loss function for training and Ω is an additional loss for enforcing sparsity. **(B)** Proposed sparsity-driven energy-based neuromorphic machine learning where L and Ω are equivalent.

From the perspective of tinyML systems, each of these family of approaches have their own shortcomings. Backpropagation (BP) has long been criticized due to issues arising from weight transport and update locking—both of which, aside from their biological implausibility, pose serious limitations for resource constrained computing platforms (Crafton et al., 2019). Weight transport problem refers to the perfect symmetry requirement between feed-forward and feedback weights in backpropagation, making weight updates non-local and requiring each layer to have complete information about all weights from downstream layers. This reliance on global information leads to significant energy and latency overheads in hardware implementations. Update locking implies that backpropagation has to wait for a full forward pass before weight updates can occur in the backward pass, causing high memory overhead due to the necessity of buffering inputs and activations corresponding to all layers. On the other hand, neuromorphic algorithms relying on local learning rules do not require global information and buffering of intermediate values for performing weight updates. However, these algorithms are not optimized w.r.t. a network objective, and it is difficult to interpret their dynamics and fully optimize the network parameters for solving a certain task. Additionally, neither of these existing approaches inherently incorporates optimization for sparsity within the learning framework. This is an important aspect for tinyML systems, because similar to biological systems (Attwell and Laughlin, 2001), generation and transmission of spike information from one part of the network to the other consumes the maximum amount of power in neuromorphic systems (Sorbaro et al., 2020). Even though Time-to-First-Spike (TTFS) SNNs (Mostafa, 2017; Zhou et al., 2021) have been shown to be sparser and more energy-efficient in comparison to their rate-coded counterparts, these networks still use backpropagation using non-spiking variables (floating-point numbers) and as such inherit the limitations of BP-based approaches when used in the context of tinyML applications. In absence of a direct control over sparsity, energy-efficiency in neuromorphic machine learning has largely been a secondary consideration, achieved through external constraints on network connectivity and/or quantization level of its neurons and synapses (Esser et al., 2016), or through additional penalty terms that regularize some statistical measure of spiking activity like firing rates (Neil et al., 2016), or the total number of synaptic operations (Sorbaro et al., 2020). This is illustrated in Figure 1A where finding optimal weight parameters w^*^ for a given task is then equivalent to finding a solution that simultaneously minimizes a training loss L and a second cost function Ω which favors sparser solutions, with the relative importance being determined by a regularization hyper-parameter β.

In order to truly exploit neuromorphic principles for tinyML architectures, we would need to design energy-based learning models that are also neurally relevant or backpropagation-less and at the same time enforce sparsity in the network's spiking activity. Over the last few years, there has been a growing interest in developing algorithms for training neural networks that overcomes one or more constraints of the backpropagation algorithm. One well-known method is feedback alignment—also known as random backpropagation—which eradicates the weight transport problem by using fixed random weights in the feedback path for propagating error gradient information (Liao et al., 2016; Lillicrap et al., 2016). Subsequent research showed that directly propagating the output error (Nøkland and Eidnes, 2019) or even the raw one-hot encoded targets (Frenkel et al., 2021) is sufficient to maintain feedback alignment, and in case of the latter, also eradicates update locking by allowing simultaneous and independent weight updates at each layer. Equilibrium propagation (Scellier and Bengio, 2017) is another biologically relevant algorithm for training energy-based models, where the network initially relaxes to a fixed-point of its energy function in response to an external input. In the subsequent phase when the corresponding target is revealed, the output units are nudged toward the target in an attempt to reduce prediction error, and the resulting perturbations rippling backward through the hidden layers were shown to contain error gradient information akin to backpropagation. Yet another class of algorithms are predictive coding frameworks, which use local learning rules to hierarchically minimize prediction errors (Whittington and Bogacz, 2017; Millidge et al., 2020). However, it is not clear how we can design such systems within a neuromorphic tinyML framework which can (a) generate spiking responses within an energy-based model; (b) learn optimal parameters for a given task using local learning rules; and (c) additionally optimize itself for sparsity such that it is able to encode the solution with the fewest number of spikes possible without relying on additional regularizing terms.

In this work we propose a framework for designing neuromorphic tinyML systems that is backpropagation-less but is also able to enforce sparsity in network spiking activity in addition to conforming to additional structural or connectivity constraints imposed on the network. The framework builds upon our previously proposed spiking neuron and population model based on a Growth Transform dynamical system (Gangopadhyay et al., 2020), where the dynamical and spiking responses of a neuron are derived directly from an energy functional of continuous-valued neural variables (membrane potentials). This provides the model with enough granularity to independently control different neuro-dynamical parameters, e.g., the shape of action potentials or transient population dynamics like bursting, spike frequency adaptation, etc. However, in the previous framework (Gangopadhyay et al., 2020), the model had a fixed energy landscape with constant synaptic weights. In this work we extend the framework to incorporate learning or synaptic adaptation, which will play a pivotal role in determining the optimal network configuration. Specifically, in this paper we address learning/adaptation to reshape the energy landscape optimally for solving standard machine learning tasks. We moreover show how we can exploit the inherent dynamics of Growth Transform neurons to design networks where learning the optimal parameters for a learning task simultaneously minimizes an important energy metric for the system—the sum-total of spiking activity across the network. This is illustrated in Figure 1B, where the energy function for reducing the training error also represents the network-level spiking activity, such that minimizing one is equivalent to minimizing the other. Moreover, since the energy functionals for deriving the optimal neural responses as well as weight parameters are directly expressible in terms of continuous-valued membrane potentials, the Growth Transform (GT) neuron model can implement energy-based learning using the neural variables themselves, without requiring to resort to rate-based representations, spike-time conversions, output approximations, or the use of external classifiers. Additionally, we present a multi-layered network architecture where lateral connections within a layer allow each layer to learn a non-linear encoding of its input, whereas the connections between layers could remain static. This allows us to design networks where weight adaptation only happens between neurons on the same layer, which could be locally implemented on hardware. We also show in this paper that the sparsity constraints on the network's spiking activity acts as a regularizer that improves the Growth Transform neural network's (GTNN's) generalization performance when learning with few training samples (few-shot learning).

The paper is organized as follows. Section 2 derives the energy function for minimizing the average power dissipation in a generic neuron model under specified constraints, and shows how spike generation can be framed as a constraint violation in such a network. We show that the energy function has the same form as the network objective we derived for the spiking neuron and population model in Gangopadhyay et al. (2020), and could therefore be optimized using the continuous-time Growth Transform dynamical system presented previously. We exploit the properties of GT neurons to design a differential network configuration consisting of ON-OFF neuron pairs which always satisfies a linear relationship between the input and response variables. We then present a learning framework which adapts weights in the network such that the linear relationship is satisfied with the highest network sparsity possible, i.e., the minimum number of spikes elicited across the network. In section 3, we show how appropriate choices of network architectures enable us to solve standard unsupervised and supervised machine learning tasks using the GT network, while simultaneously optimizing for sparsity. We further extend the previous results to solve non-linearly separable classification problems using three different end-to-end spiking networks with progressively increasing flexibility in training (and consequently, sparsity), and present the results for few-shot and one-shot learning on a benchmark machine olfaction dataset, as well as results on other benchmark datasets. Finally, section 4 concludes the paper with a discussion about the scope of future research in this area.

## 2. Materials and Methods

Throughout the paper we will conform to the mathematical notations summarized below. Also, unless specified all quantities considered in the methods will be unit-less or in the form of ratios.

**Table:** 

**Notations**	
*x*	Real scalar variable
***x***	Real-valued vector with *x*_*i*_ as its *i*-th element
***X***	Real-valued matrix with *X*_*ij*_ as the element at the *i*-th row and the *j*-th column
*x*_*i*_(*t*)	*i*-th element of real-valued vector ***x*** at time *t*
$\bar{x}\left(t\right)$	Empirical expectation of the time-varying signal *x*(*t*) estimated over an
	asymptotically infinite window, i.e., $\underset{T\to \infty }{\lim}\frac{1}{T}\int_{0}^{T}x\left(t\right)dt$
ℝ^*M*^	Vector space spanned by *M*-dimensional real vectors
|*x*|	Absolute value of a scalar
||***x***||_*p*_	*l*_*p*_-norm of an *M*-dimensional vector, defined as ${\left(\overset{M}{\underset{i=1}{\sum }}|x_{i}{|}^{p}\right)}^{1/p}$
***x*** ^*T*^	Transpose of the vector ***x***
$\frac{\partial H}{\partial x}$	Gradient vector ${\left[\frac{\partial H}{\partial x_{1}},\frac{\partial H}{\partial x_{2}},...,\frac{\partial H}{\partial x_{M}}\right]}^{T}$

### 2.1. Spike Generation Viewed as A Constraint Violation

**Figure 2:**
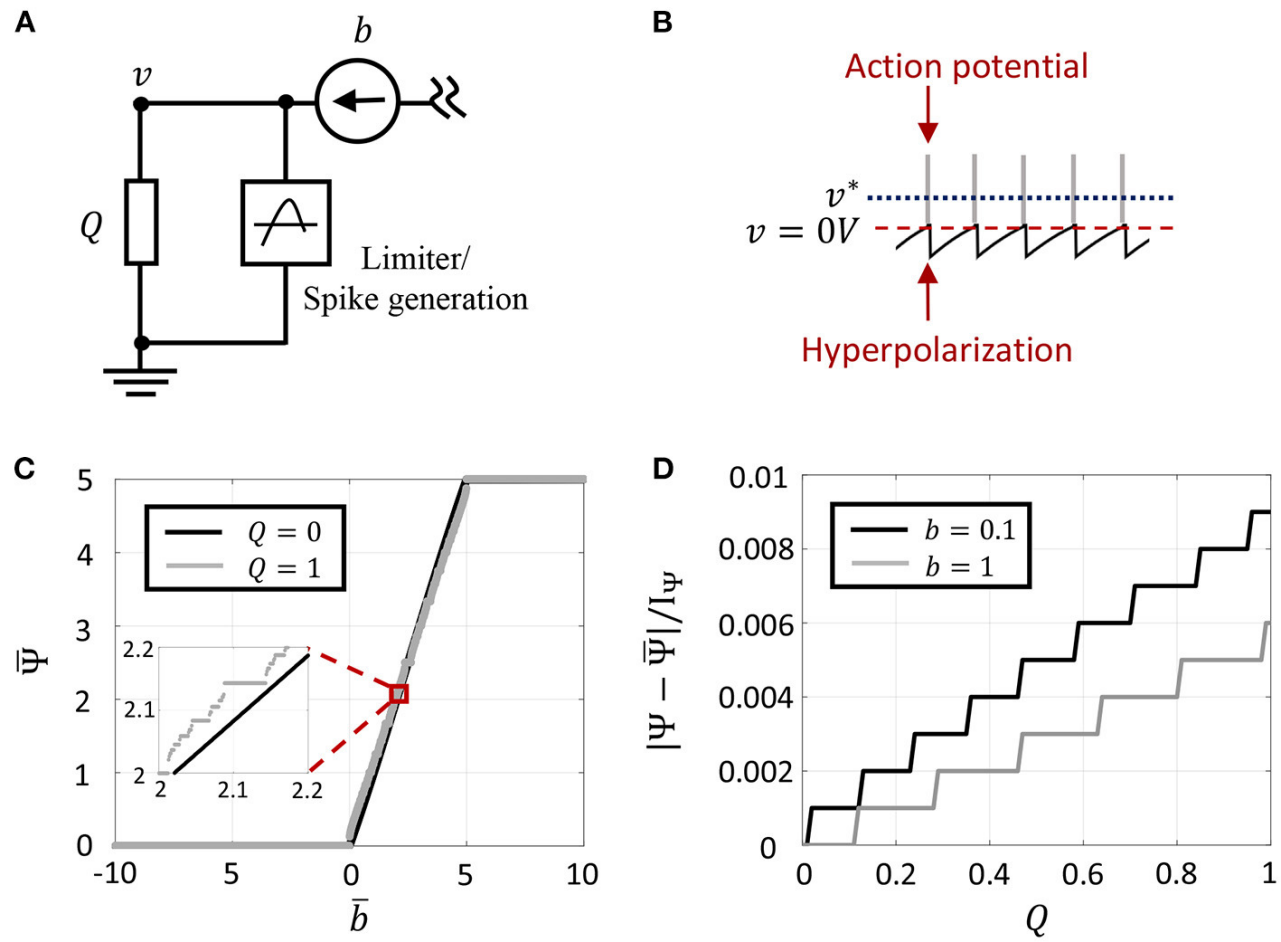
**(A)** Circuit model for a single neuron model with external current input. **(B)** Oscillatory dynamics in GT neuron model when the optimal solution v^*^ goes above the spike threshold, and the composite spike signal upon addition of spikes. **(C)** Plot of $\bar{\Psi }$ vs. $\bar{b}$, for two different values of *Q*. **(D)** Error introduced as a result of approximating Ψ by $\bar{\Psi }$ for different values of Q and two different current inputs.

Consider a general circuit model of a single neuron as shown in Figure 2A, whose intra-cellular membrane potential is denoted by *v* ∈ ℝ. It receives an external stimulus as current input *b*. Then, the average power *P* ∈ ℝ dissipated by the neuron is given by

(1) $$\begin{array}{l} P=\frac{1}{2}Qv^{2}-bv, \end{array}$$

where *Q* ∈ ℝ^+^ captures the effect of leakage impedance, as shown in Figure 2A. We will impose biophysical constraints that the membrane potential *v* be bounded as

(2) $$\begin{array}{l} -v_{c}\leq v\leq 0, \end{array}$$

where *v*_*c*_ > 0 is a constant potential acting as a lower-bound, and 0 is a reference potential acting as a threshold voltage. Minimizing the average power dissipation of the neuron under the bound constraint in (2) is equivalent to solving the following optimization problem

(3) $$\begin{array}{l} \underset{-v_{c}\leq v\leq 0}{\min}P=\underset{-v_{c}\leq v\leq 0}{\min}\frac{1}{2}Qv^{2}-bv. \end{array}$$

Let Ψ ≥ 0 be the KKT (Karush-Kuhn-Tucker) multiplier corresponding to the inequality constraint *v* ≤ 0, then the optimization in (3) is equivalent to

(4) $$\begin{array}{l} \underset{|v|\leq v_{c},\Psi }{\min}H(v)=\underset{|v|\leq v_{c},\Psi }{\min}\frac{1}{2}Qv^{2}-bv+\Psi v \end{array}$$

where Ψ ≥ 0, and Ψv^*^ = 0 satisfy the KKT complementary slackness criterion for the optimal solution v^*^ (Kuhn and Tucker, 1951). The solution to the optimization problem in (4) satisfies the following first-order condition

(5) $$\begin{array}{l} \;\Psi =-Qv^{*}+b\\ \Psi v^{*}=0;\Psi \geq 0;|v^{*}|\leq v_{c}. \end{array}$$

The first-order condition in (5) could be extended to time-varying input *b*(*t*) where (5) can be expressed in terms of the temporal expectation (defined in the Notations table) of the optimization variables as

(6) $$\begin{array}{l} \;\bar{\Psi }\approx -Q\bar{v}+\bar{b}\\ \Psi v=0;\Psi \geq 0;|v|\leq v_{c}. \end{array}$$

Note that here the KKT constraints Ψ*v* = 0;Ψ ≥ 0 need to be satisfied for all instantaneous values and at all times, and not only at the optimal solution v^*^. Thus, Ψ acts as a switching function which results from the violation of the constraint *v* ≤ 0. In the next section we show that a dynamical system with a specific form of Ψ(.) and satisfying the KKT constraints, naturally defines the process of spike-generation.

### 2.2. Growth Transform Neuron Model

One way to satisfy the first-order conditions (6) using a dynamical systems approach would be to define Ψ as a barrier function

(7) $$\begin{array}{l} \Psi =\left\{\begin{array}{ccc} I_{\Psi } & ; & v>0\\ 0 & ; & v\leq 0 \end{array}\right\}. \end{array}$$

with *I*_Ψ_ ≥ 0 denotes a hyperpolarization parameter. Such a barrier function ensures that the complementary slackness condition holds at all times, and as we show later, the temporal expectation (as defined in Notations) $\bar{\Psi }\to \Psi$ in the limit as *Q* → 0. For the form of the spike function in (7), we can write

(8) $$\begin{array}{l} \Psi v=\overset{v}{\underset{-\infty }{\int }}\Psi \left(\eta \right)d\eta . \end{array}$$

Thus the optimization problem in (9) can be rewritten as

(9) $$\begin{array}{l} \underset{|v|\leq v_{c}}{\min}H(v)=\underset{|v|\leq v_{c}}{\min}\frac{1}{2}Qv^{2}-bv+\overset{v}{\underset{-\infty }{\int }}\Psi \left(\eta \right)d\eta . \end{array}$$

The cost function H can be optimized under the bound constraints |*v*| ≤ *v*_*c*_ using a dynamical systems approach like the Growth Transform (GT) neuron model (Gangopadhyay et al., 2020). The mathematical derivation and the properties of the GT neuron model can be found in Gangopadhyay et al. (2020) and here we summarize the model for the sake of completeness. For the GT neuron, the membrane potential *v* evolves according to the following first-order non-linear differential equation

(10) $$\begin{array}{l} \tau \left(t\right)\frac{dv}{dt}+v=v_{c}\frac{-gv_{c}+\lambda v}{-gv+\lambda v_{c}}, \end{array}$$

where

(11) $$\begin{array}{l} g=Qv-b+\Psi . \end{array}$$

Here λ is a fixed hyper-parameter that is chosen such that λ > |*g*|, and 0 ≤ τ(*t*) < ∞ is a modulation function that can be tuned individually for each neuron and models the excitability of the neuron to external stimulation. It was shown in Gangopadhyay et al. (2020) that for non-saturating neurons with responses −*v*_*c*_ < *v* (which includes spiking neurons as well as non-spiking neurons that do not cross the threshold), Equation (10) evolves the dynamical system such that H is minimized, irrespective of the choice of τ(*t*). Figure 2B shows a sample membrane potential and spike function trace of a single GT neuron in response to a fixed stimulus. The neuron model has a spiking threshold at 0 V, which corresponds to the discontinuity in the spike function Ψ(.). When the neuron receives a positive input, the optimal solution, v^*^, shifts above the threshold to a level that is a function of the stimulus magnitude. When *v* tries to exceed the threshold in an attempt to reach the optimal solution, Ψ(.) penalizes the energy functional, forcing *v* to reset below the threshold. The stimulus and the barrier function introduce opposing tendencies as long as the stimulus is present, making *v* oscillate back and forth around the discontinuity (spike threshold). During the brief period when *v* reaches the threshold, we assume that the neuron enters into a runaway state leading to a voltage spike, shown by gray bars in the figure. In Gangopadhyay et al. (2020), we showed that the modulation function τ(.) in (10) provides another degree of freedom that can be varied to model different transient firing statistics based on local and/or global variables. For example, it was shown how the modulation function can be varied based on local variables like membrane potentials or local firing rates to reproduce single-neuron response characteristics like tonic spiking, bursting, spike-frequency adaptation, etc., or based on global properties like the state of convergence of the network to yield different population-level dynamics.

#### 2.2.1. Orthogonal, Mixed-Signal, and ReLU Encoding of A Single GT Neuron

The GT neuron model satisfies the following first order condition

(12) $$\begin{array}{l} \;\bar{\Psi }\approx -Q\bar{v}+\bar{b}\\ \Psi v=0;\Psi \geq 0;|v|\leq v_{c}. \end{array}$$

Then as *Q* → 0,

(13) $$\begin{array}{l} \bar{\Psi }\to \text{ReLU}(\bar{b}), \end{array}$$

where

(14) $$\begin{array}{l} \text{ReLU}\left(z\right)=\left\{\begin{array}{c} z\;;\;z>0\\ 0\;;\;z\leq 0 \end{array}\right\}. \end{array}$$

Figure 2C demonstrates (13) for two different values of *Q*. Since *I*_Ψ_ also controls the refractory period of the GT neuron and the temporal expectation is computed over a finite time window, there exists an upper-bound on the maximum firing rate as shown in Figure 2C. Note that $\bar{\Psi }$ corresponds to an averaging over discrete events, thus the result (13) exhibits quantization effects. But in the limit *Q* → 0, $\bar{\Psi }$ converges toward the floating-point solution Ψ in (5). This is demonstrated in Figure 2D which plots the absolute difference between Ψ and $\bar{\Psi }$ (normalized by *I*_Ψ_) for different values of the leakage impedance. Note that the quantization step of 0.001 in this plot arises due to a finite number of spikes that can occur within a finite window size (1,000 time-steps for the plot shown). This quantization error could be further reduced by considering a larger window size. For the rest of this paper, we will use the variables Ψ, *v* and their temporal expectation $\bar{\Psi },\bar{v}$ interchangeably with the understanding that they converge toward each other in the limit *Q* → 0. This also implies we will interchangeably use optimizations (4) and (9) to understand the asymptotic properties of the solution.

An interesting observation about the response of a single GT neuron from the first-order condition in (6) is

(15) $$\begin{array}{l} \Psi +Qv=b \end{array}$$

(16) $$\begin{array}{l} \Psi v=0. \end{array}$$

Thus, all GT neurons are encoding the input stimuli as two orthogonal components, one being the discrete (or digital) spike-function Ψ and the other being the analog membrane potential *v*. We extend this concept in the next section to a network of ON-OFF neurons that can produce coupled orthogonal trajectories.

#### 2.2.2. An ON-OFF GT Neuron Model for Stimulus Encoding

**Figure 3:**
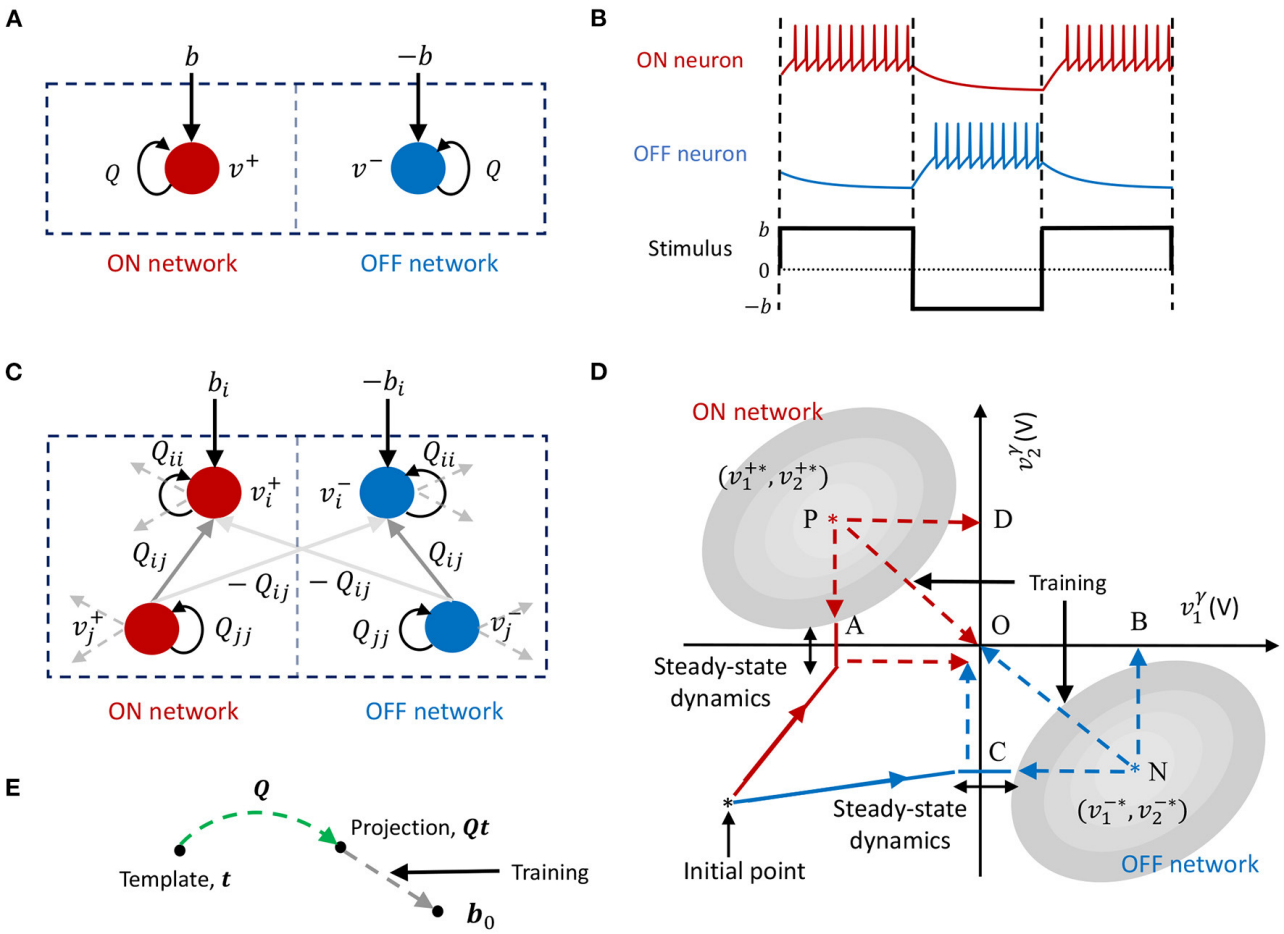
**(A)** An ON-OFF neuron pair. **(B)** Temporal dynamics of an ON-OFF neuron pair in response to positive and negative stimuli. **(C)** Coupled differential GT network. **(D)** Firing rate minimization through weight adaptation in a coupled differential network. **(E)** Template projection using a GT network.

The fundamental building block in the proposed GTNN learning framework is an ON-OFF GT neuron model which we describe here. Consider a GT network as shown in Figure 3A, comprising of a pair of neurons, an ON neuron and an OFF neuron. We will denote the respective membrane potentials of the ON neuron and OFF neuron as *v*^+^ and *v*^−^. The external input *b* is presented differentially to the neuron pair, where the ON neuron receives a positive input stimulus *b* and the OFF neuron receives a negative input stimulus −*b*. We will assume for now that the neurons do not have any synaptic projections to each other, as shown in the Figure 3A. The optimization problem (4) decomposes into two uncoupled cost functions corresponding to the ON and OFF neurons, respectively as

(17) $$\begin{array}{l} \underset{|v^{+}|\leq v_{c}}{\min}H(v^{+})=\underset{|v^{+}|\leq v_{c}}{\min}\frac{1}{2}Q{v^{+}}^{2}-bv^{+}+\Psi^{+}v^{+},\text{ and} \end{array}$$

(18) $$\begin{array}{l} \underset{|v^{-}|\leq v_{c}}{\min}H(v^{-})=\underset{|v^{-}|\leq v_{c}}{\min}\frac{1}{2}Q{v^{-}}^{2}+bv^{-}+\Psi^{-}v^{-} \end{array}$$

This corresponds to the following first-order conditions for the differential pair

(19) $$\begin{array}{l} Qv^{+}+\Psi^{+}=b,\text{ and} \end{array}$$

(20) $$\begin{array}{l} Qv^{-}+\Psi^{-}=-b, \end{array}$$

along with the non-negativity and complementary conditions for the respective spike functions as

(21) $$\begin{array}{l} \Psi^{+}\geq 0;\Psi^{+}v^{+}=0,\text{ and}\\ \Psi^{-}\geq 0;\Psi^{-}v^{-}=0. \end{array}$$

Let us consider two cases based on the sign of the input *b*.

*Case 1*. *b* ≥ 0: When *b* is positive, we obtain the following set of solutions to (19)–(20) under the above constraints

(22) $$\begin{array}{l} v^{+}=0,\Psi^{+}=b,\text{ and} \end{array}$$

(23) $$\begin{array}{l} Qv^{-}=-b,\Psi^{-}=0. \end{array}$$

*Case 2*. *b* < 0: When *b* is negative, the corresponding solutions are as follows

(24) $$\begin{array}{l} Qv^{+}=b,\Psi^{+}=0,\text{ and} \end{array}$$

(25) $$\begin{array}{l} v^{-}=0,\Psi^{-}=-b. \end{array}$$

Combining the two cases, the ON-OFF variables *v*^+^, *v*^−^ satisfy the following important properties:

(26) $$\begin{array}{l} v^{+}v^{-}=0, \end{array}$$

(27) $$\begin{array}{l} Q(v^{+}-v^{-})=\Psi^{+}-\Psi^{-}=b \end{array}$$

(28) $$\begin{array}{l} \Psi^{+}+\Psi^{-}=-Q(v^{+}+v^{-}). \end{array}$$

Property (26) show that the membrane voltages *v*^+^ and *v*^−^ are always orthogonal to each other. Figure 3B shows that the orthogonality also holds for their respective temporal dynamics as well when the input stimulus is turned ON and OFF. This temporal ON-OFF responses have been well documented for networks of biological neurons as well (Hubel and Wiesel, 1962; Behnia et al., 2014; Moustafa, 2015; Saha et al., 2017). Property (27) show that the ON-OFF neurons together faithfully encode the input stimuli. In this regard, the pair behaves as an analog-to-digital converter (ADC) which maps the time-varying analog input into a train of output binary spikes. Property (28) in conjunction with Property (26) leads to

(29) $$\begin{array}{l} \left(\Psi^{+}+\Psi^{-}\right)=-Q\left(v^{+}+v^{-}\right) \end{array}$$

(30) $$\begin{array}{l} =Q|v^{+}-v^{-}| \end{array}$$

which states that the average spiking rate of an ON-OFF network encodes the norm of the differential membrane potential *v* = *v*^+^ − *v*^−^. This property has been used in the next section to simultaneously enforce sparsity and solve a learning task.

### 2.3. A Sparsity-Driven Learning Framework to Adapt **Q**

We now extend the ON-OFF neuron pair to a generic network comprising *M* neuron pairs, as shown in Figure 3C. The *i*-th neuron pair is coupled differentially to the *j*-th neuron pair through a trans-conductance synapse denoted by its weight *Q*_*ij*_ ∈ ℝ. The differential stimuli to the ON-OFF network in (19)–(20) can be generalized to comprise the external input *b*_*i*_ and inputs from other ON-OFF neuron pairs as

(31) $$\begin{array}{l} b_{i}-\underset{j\neq i}{\sum }Q_{ij}\left(v_{j}^{+}-v_{j}^{-}\right). \end{array}$$

Denoting the *Q* in (19)–(20) by *Q*_*ii*_ leads to the first-order conditions for the *i*-th ON-OFF neuron pair as

(32) $$\begin{array}{l} Q_{ii}v_{i}^{+}+\Psi_{i}^{+}=-\underset{j\neq i}{\sum }Q_{ij}\left(v_{j}^{+}-v_{j}^{-}\right)+b_{i},\text{ and} \end{array}$$

(33) $$\begin{array}{l} Q_{ii}v_{i}^{-}+\Psi_{i}^{-}=\underset{j\neq i}{\sum }Q_{ij}\left(v_{j}^{+}-v_{j}^{-}\right)-b_{i}. \end{array}$$

As before, each neuron in the network satisfies

(34) $$\begin{array}{l} Q_{ii}(v_{i}^{+}-v_{i}^{-})=b_{i}-\underset{j\neq i}{\sum }Q_{ij}\left(v_{j}^{+}-v_{j}^{-}\right),\text{ or} \end{array}$$

(35) $$\begin{array}{l} \overset{M}{\underset{j=1}{\sum }}Q_{ij}\left(v_{j}^{+}-v_{j}^{-}\right)=b_{i}, \end{array}$$

Equation (35) can be written in a matrix form as a linear constraint

(36) $$\begin{array}{l} Qv=b, \end{array}$$

where $Q=\left\{Q_{ij}\right\}\in {\mathbb{R}}^{M}\times {\mathbb{R}}^{M}$ is the synaptic matrix, $v=\left\{v_{i}^{+}-v_{i}^{-}\right\}\in {\mathbb{R}}^{M}$ is the ON-OFF membrane potential vector and $b=\left\{b_{i}\right\}\in {\mathbb{R}}^{M}$ is the input stimuli vector. Note that the linear constraint (36) arose as a result of each neuron optimizing its local power dissipation as

(37) $$\begin{array}{l} \underset{v^{+},v^{-}}{\min}H\left(v^{+}\right)+H\left(v^{-}\right), \end{array}$$

with the synaptic connections given by ***Q***. In addition to each of the neurons minimizing its respective power dissipation with respect to the membrane potentials $v_{i}^{+},v_{i}^{-}$, the total spiking activity of the network could be minimized with respect to ***Q*** as

(38) $$\begin{array}{l} \underset{Q}{\min}L\left(Q\right)=\underset{Q}{\min}\overset{M}{\underset{i=1}{\sum }}(\Psi_{i}^{+}+\Psi_{i}^{-}). \end{array}$$

From (30),

(39) $$\begin{array}{l} L\left(Q\right)=\overset{M}{\underset{i=1}{\sum }}Q_{ii}|v_{i}^{+}-v_{i}^{-}|\\ \;=||\Lambda v{||}_{1}, \end{array}$$

where Λ ∈ ℝ^*M*^ × ℝ^*M*^ is a diagonal matrix with Λ_*ii*_ = *Q*_*ii*_; Λ_*ij*_ = 0 ∀*i* ≠ *j*. Hence, solving the optimization problems in (37) and (38) simultaneously is equivalent to solving the following *L*_1_ optimization

(40) $$\begin{array}{l} \underset{Q}{\min}||\Lambda v{||}_{1} \end{array}$$

(41) $$\begin{array}{l} \text{s.t. }Qv=b. \end{array}$$

Note that the *L*_1_ optimization bears similarity to compressive sensing formulations (Candès and Wakin, 2008). However, in this case, the objective is to find the sparsest membrane potential vector by adapting the synaptic weight matrix ***Q*** in a manner that the information encoded by the input stimuli is captured by the linear constraint in (41). This rules out the trivial sparse solution v^*^ = 0 for a non-zero input stimuli. A gradient descent approach is applied to the cost-function L(.) in (38) to update the synaptic weight *Q*_*ij*_ according to

(42) $$\begin{array}{l} \Delta Q_{ij}=-\eta \frac{\partial L}{\partial Q_{ij}}, \end{array}$$

where η > 0 is the learning rate. Using the property (13), one obtains the following spike-based local update rule

(43) $$\begin{array}{l} \Delta Q_{ij}=\eta \left(\Psi_{i}^{+}(v_{j}^{+}-v_{j}^{-})-\Psi_{i}^{-}\left(v_{j}^{+}-v_{j}^{-}\right)\right) \end{array}$$

(44) $$\begin{array}{l} =\eta (\Psi_{i}^{+}-\Psi_{i}^{-})(v_{j}^{+}-v_{j}^{-}),i\neq j. \end{array}$$

Note that since there are no synaptic connections between the ON-OFF neurons of the same pair,

(45) $$\begin{array}{l} \Delta Q_{ii}=\eta (\Psi_{i}^{+}v_{i}^{+}+\Psi_{i}^{-}v_{i}^{-})=0, \end{array}$$

implying that the self-connections in GTNN do not change during the adaptation. This feature is important because *Q*_*ii*_ can be adapted independently to adjust the precision of the approximation in (13). For the rest of the paper, we will fix *Q*_*ii*_ = 1 so that Λ is an identity matrix. Also, note that the synaptic matrix ***Q*** need not be symmetric which makes the framework more general than conventional energy-based optimization.

Figure 3D pictorially depicts how the sparsest solution is achieved through firing rate minimization for a differential network where *M* = 2. We assume for this exposition that the matrix ***Q*** is symmetric and hence the solution can be visualized using energy contours. The figure shows energy contours in absence of the barrier function for the positive and negative parts of the network superimposed on the same graph. The corresponding optimal solutions $\left(v_{1}^{+*},v_{2}^{+*}\right)$ and $\left(v_{1}^{-*},v_{2}^{-*}\right)$, denoted by P and N, respectively, satisfy $v_{i}^{+*}=-v_{i}^{-*},i=1,2$. As discussed previously, the presence of the barrier function prohibits the membrane potentials from reaching the optimal solutions. Instead, the membrane potentials exhibit steady-state spiking dynamics around the spike thresholds. These steady-state dynamics corresponding to the positive and negative networks are shown in the figure as lines at points A and C where the two coupled networks breach the spiking threshold under the respective energy contours in steady-state.

During weight adaptation since $\Psi_{i}^{+}$ is minimized ∀*i*, network weights evolve such that the membrane potentials breach the spiking threshold less often, which essentially pushes the optimal solution for the positive network toward A. However, since the two networks (and hence their solutions) are differential, the optimal solution for the negative network is pushed toward B. Similarly during the weight adaptation process since $\Psi_{i}^{-}$ is also minimized ∀*i*, optimal solution for the negative network is pushed toward C such that its own spike threshold constraints are violated less frequently, which in turn pushes the optimal solution for the positive network toward D. The positive network therefore moves toward the path $\vec{P0}$ given by the vector sum of paths $\vec{PD}$ and $\vec{PA}$. Similarly, the negative network moves toward the path $\vec{NO}$, given by the vector sum of paths $\vec{NC}$ and $\vec{NB}$. This minimizes the overall firing rate of the network and drives the membrane potentials of each differential pair toward zero, while simultaneously ensuring that the linear constraint in (36) is always satisfied.

#### 2.3.1. Linear Projection Using Sparse GT Network

The *L*_1_ optimization framework described by (40) provides a mechanism to synthesize and understand the solution of different variants of GTNN. For instance, if input stimulus vector ***b*** is replaced by

(46) $$\begin{array}{l} b=b_{0}-Qt. \end{array}$$

where ***t*** ∈ ℝ^*M*^ is a fixed template vector then according to (40), the equivalent *L*_1_ optimization leads to

(47) $$\begin{array}{l} \underset{Q}{\min}||v{||}_{1}\text{ s.t. }Qv=b_{0}-Qt. \end{array}$$

The *L*_1_ optimization chooses the solution ***Qt*** = ***b*_0_** such that ||***v***||_1_ → 0. Thus,

(48) $$\begin{array}{l} \underset{Q}{\min}||v{||}_{1}\Rightarrow \underset{Q}{\min}||b_{0}-Qt{||}_{1}. \end{array}$$

The synaptic update rule corresponding to the modified loss function is given by

(49) $$\begin{array}{l} \Delta Q_{ij}=\eta (\Psi_{i}^{+}-\Psi_{i}^{-})(v_{j}^{+}-v_{j}^{-}+t_{j}). \end{array}$$

This is depicted in Figure 3E, which shows that the projection of the template vector, ***Qt***, evolves toward ***b***_0_ with synaptic adaptation. In section 3, we show how this could be used to solve unsupervised learning problems like domain description and anomaly detection.

#### 2.3.2. Inference Using Network Sparsity

The sparsity in network spiking activity could directly be used for optimal inference. The rationale is that the *L*_1_ optimization in (40) and (47) chooses the synaptic weights ***Q*** that exploits the dependence (statistical or temporal) between the different elements of the stimulus vector ***b*** to reduce the norm of membrane potential vector ||***v***||_1_ and hence the spiking activity. Thus, the process of inference involves choosing the stimulus that produces the least normalized network spiking activity defined as

(50) $$\begin{array}{l} \underset{b}{arg min}\rho_{b}=\frac{1}{2M}\overset{M}{\underset{i=1}{\sum }}\left(s_{b_{i}}^{+}+s_{b_{i}}^{-}\right), \end{array}$$

where *M* denotes the total number of differential pairs in the network and $s_{b_{i}}^{+}$, $s_{b_{i}}^{-}$ are the average spike counts of the *i*-th ON-OFF pair, given by $s_{b_{i}}^{\gamma }=\Psi_{b_{i}}^{\gamma }/I_{\Psi },\gamma =+,-$, when the stimulus ***b*** is presented as input.

## 3. Results

In this section, we apply the learning framework introduced previously to standard machine learning tasks. We first show how different choices of neural parameters and network architectures lend themselves to solving standard unsupervised and supervised learning problems, and finally extend our findings to build end-to-end spiking networks for solving an odor recognition problem on a benchmark machine olfaction dataset.

### 3.1. Weight Adaptation Leads to Sparsity

**Figure 4:**
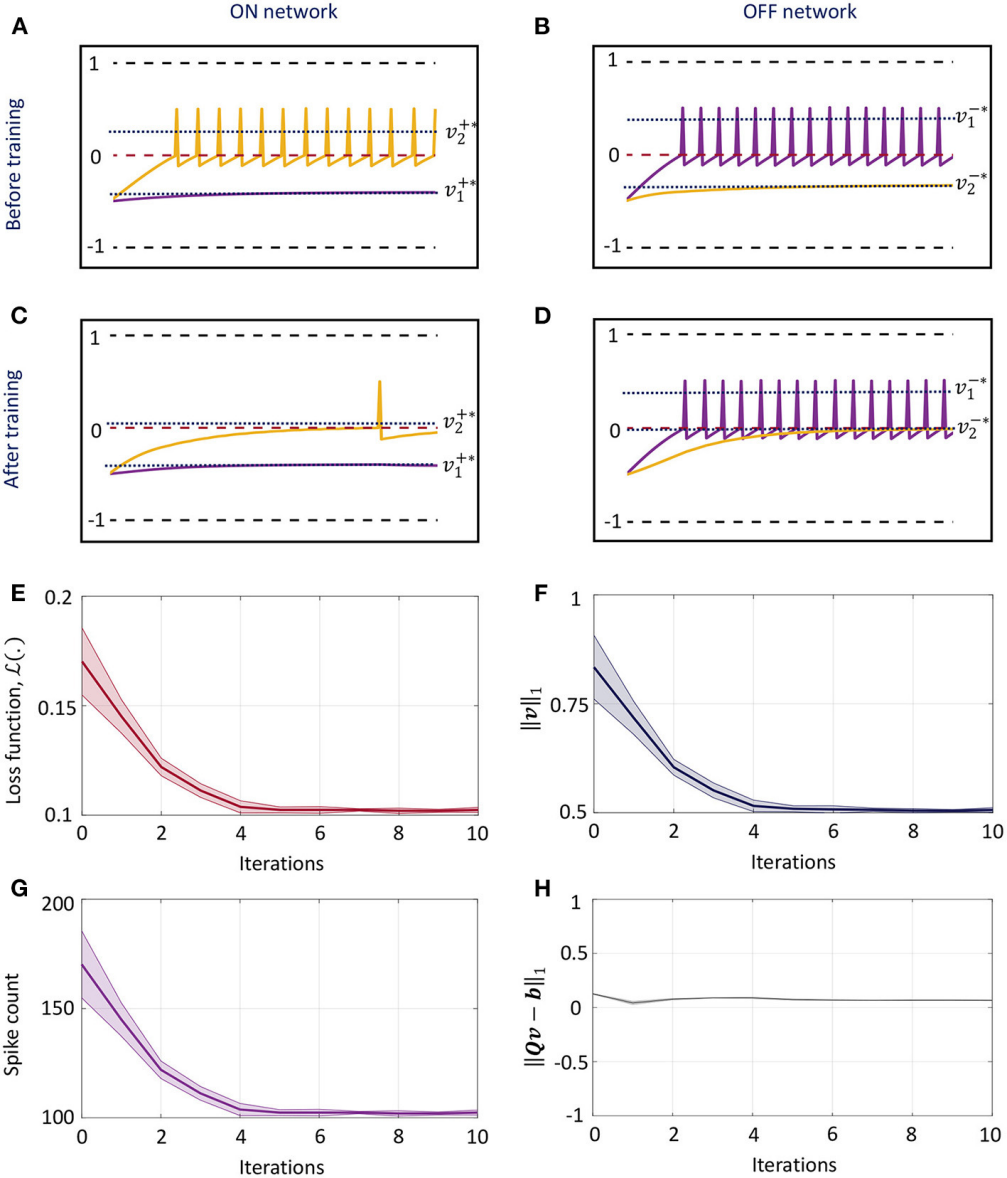
**(A,B)** Spike responses for the ON and OFF neurons respectively of a differential network with two neuron pairs for a fixed input vector before weight adaptation. **(C,D)** Corresponding plots after weight adaptation. **(E)** Evolution of the loss function with the number of training iterations for the differential network in **(A–D)** averaged over five different initial conditions. **(F–H)** Evolution of ||***v***||_1_, spike count across the network and ||***Qv*** − ***b***||_1_ with the number of training iterations for the same problem.

Figure 4 illustrates sparsity-driven weight adaptation using a differential network with two neuron pairs presented with a constant external input vector. Figures 4A,B show the spike responses corresponding to the ON and OFF networks respectively, before any weight adaptation has taken place. Figures 4C,D present the same plots post-training. Training evolves the weights such that as many elements of the vector of membrane potentials as possible can approach zero while the network still satisfies the linear constraint in (36). We see that weight adaptation is accompanied by a decline in the firing rates for neuron pair 2, while firing rates for neuron pair 1 remains largely unchanged. This is expected for a network with 2 differential pairs, since at least one neuron pair needs to spike in order to satisfy (36). Figures 4E–G plot the decrease in cost function, ||***v***||_1_ and total spike count across the network respectively as weight adaptation progresses. Figure 4H shows that ||***Qv*** − ***b***||_1_ remains close to zero throughout the training process. For Figures 4E–H, solid lines indicate mean values across five runs with different initial conditions, while the shaded regions indicate standard deviation about the mean.

### 3.2. Unsupervised Learning Using Template Projection

In this section, we formulate unsupervised machine learning tasks like domain description and anomaly detection as a template projection problem, and show how the GT network can be applied to solve them using the framework introduced in section 2.3.1. Let $x_{k}\in {\mathbb{R}}^{D},k=1,...,K$, be data points drawn independently from a fixed distribution *P*(***x***) where *D* is the dimension of the feature space, and let ***t*** ∈ ℝ^*D*^ be a fixed template vector. Then from (48), weight adaptation gives us

(51) $$\begin{array}{l} \underset{Q}{\min}L\left(Q\right)\Rightarrow \underset{Q}{\min}\frac{1}{K}\overset{K}{\underset{k=1}{\sum }}||Qt-x_{k}{||}_{1}, \end{array}$$

where L(.) has the same form as in (38). Thus, minimizing the network-level spiking activity evolves weights in the transformation matrix ***Q*** such that the projection of the template vector can represent the given set of data points with the minimum mean absolute error.

#### 3.2.1. Domain Description

In a domain description problem, we aspire to describe a set of objects or data points given by the training set so that we can faithfully distinguish them from all other data points in the vector space (Tax and Duin, 1999). Using the template projection framework introduced above, we can train a GT network to evolve toward a set of data points such that its overall spiking activity is lower for these points, indicating that it is able to describe the domain and distinguish it from others.

**Figure 5:**
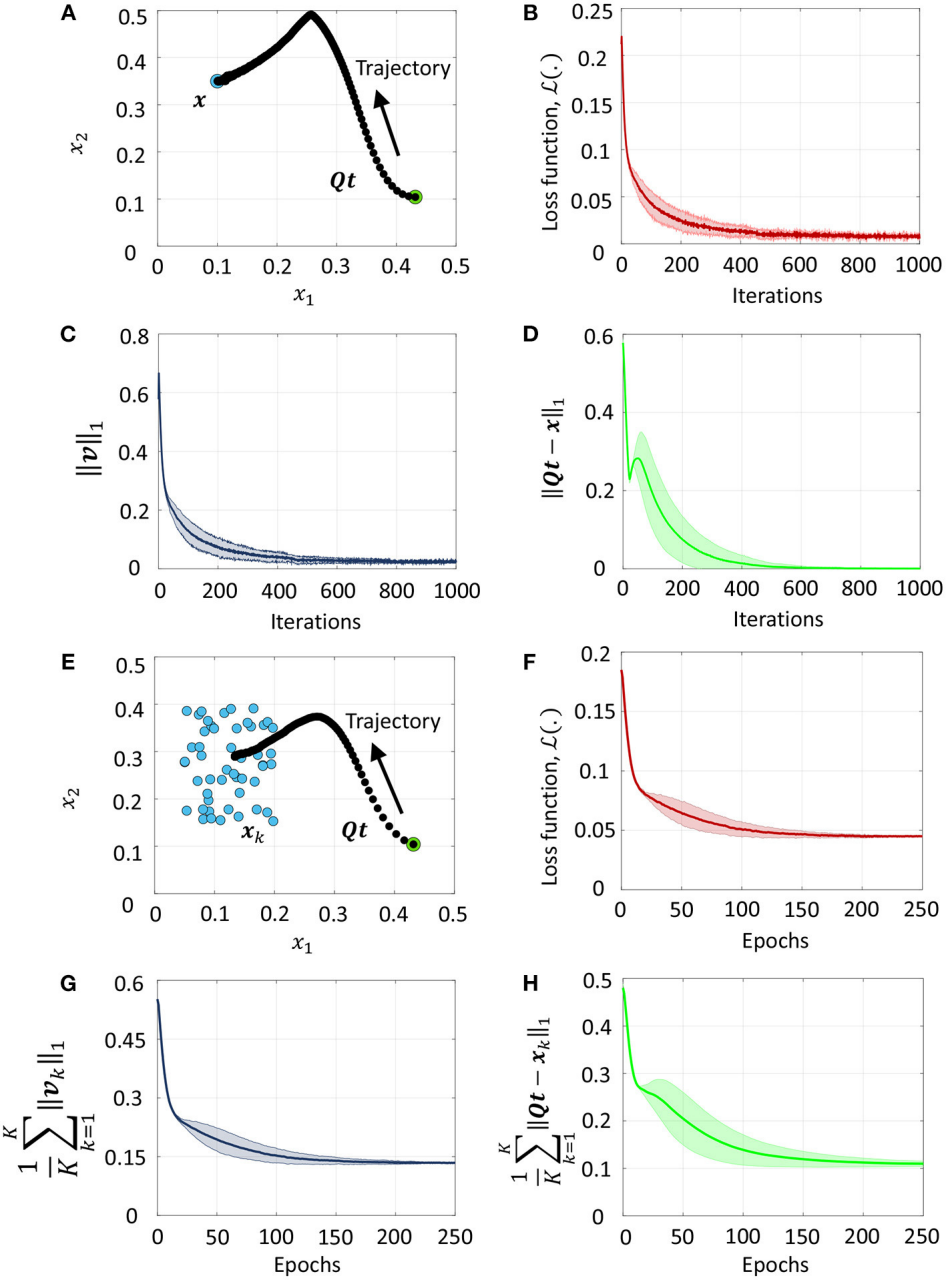
**(A)** Evolution of network trajectory with training when there is a single data point. **(B–D)** Evolution of the loss function L(.), the *L*_1_ norm of the vector of mean membrane potentials and the *L*_1_ loss respectively for the problem in **(A)** plotted against the number of training iterations. **(E)** Evolution of the network trajectory with training when there are multiple data points. **(F–H)** Plots corresponding to **(B–D)** averaged over the training dataset.

We demonstrate the equivalence between firing rate minimization across the network and the *L*_1_ loss minimization in (51) for a series of toy problems where *D* = 2. We first consider the simplest case with a single data point and a fixed threshold vector, as shown in Figure 5A. As training progresses, ***Qt*** evolves along the trajectory shown by black dots from the initial condition indicated by a green circle toward the data point indicated by a blue circle. Figure 5B plots the mean and standard deviation of the loss function L(.) for the problem in Figure 5A across 5 experiments with randomly selected initial conditions. The average loss decreases with the number of training iterations until a small baseline firing rate is reached upon convergence. Figures 5C,D plot the corresponding *L*_1_ norm of the vector of mean membrane potentials and the *L*_1_ loss in (51), respectively. We see that the *L*_1_ loss goes to zero with training, while ||***v***||_1_ approaches near zero. Figure 5E presents a case when the network is trained with multiple data points in an online manner. The network trajectory in this case evolves from the initial point to a point that lies near the median of the cluster. Figures 5F–H are the corresponding plots for the loss function, *L*_1_ norm of mean membrane potential vector and the *L*_1_ loss in (51) vs. epochs, where one epoch refers to training the network on all points in the dataset once in a sequential manner. Since here a single template vector tries to explain or approximate all data points in the cluster, the *L*_1_ loss in Figure 5H does not reach zero. However, the network adapts its weights such that it responds with the fewest number of spikes overall for the data points it sees during training, such that the network-level spiking activity is minimized for the dataset.

#### 3.2.2. Anomaly Detection

The unsupervised loss minimization framework in the preceding section drives the GT network to spike less when presented with a data point it has seen during training in comparison to an unseen data point. We can seamlessly extend this to apply to outlier or anomaly detection problems, as described in this section. When the network is trained with an unlabeled training set $x_{k}\in {\mathbb{R}}^{D},k=1,...,K$, it adapts its weights so that it fires less for data points it sees during training (or data points that are “similar” to these), referred to as members, and fires more for points that are far away (or dissimilar) to them, referred to as anomalies. Template vectors in this case are random-valued vectors held constant throughout the training procedure.

**Figure 6:**
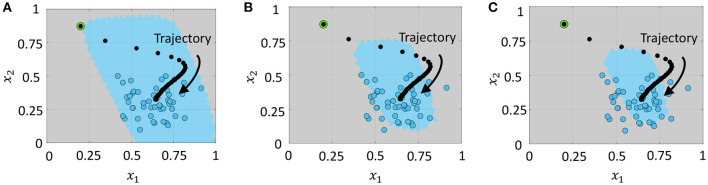
Domain described by the network when the upper bound on the number of training data points rejected as outliers is **(A)** 0%, **(B)** 25%, and **(C)** 50% of the training dataset. Blue circles indicate training data points, and blue and gray shaded regions indicate the areas identified as members and anomalies, respectively.

After training, we determine the mean firing rates of the network for each data point in the training dataset and set the maximum mean firing rate as the threshold. During inference, any data point that causes the network to fire at a rate equal to or lower than this threshold is considered to be a member, otherwise it is an outlier or anomaly. In Figure 6, blue circles correspond to the training data points. Based on the firing rate threshold computed, the network learns to classify data points similar to the training set as members (indicated by the region shaded in blue in Figure 6A, and others as anomalies (indicated by the region shaded in gray). We can also tune the firing rate threshold appropriately in order to reject a pre-determined fraction of training data points as outliers. Figures 6B,C show the contraction in the domain described by the anomaly detection network when the threshold was progressively reduced to reject 25 and 50% of the training data points as outliers.

### 3.3. Supervised Learning

In this section, we exploit the framework outlined in (40) to design networks that can solve linear classification problems using the GT network. Consider a binary classification problem given by the training dataset (***x***_*k*_, *y*_*k*_), *k* = 1, ..., *K*, drawn independently from a fixed distribution *P*(***x**, y*) defined over ℝ^*D*^ × {−1, +1}. The vector ***x***_*k*_ is denoted as the *k*-th training vector and *y*_*k*_ is the corresponding binary label indicating class membership (+1 or -1). We consider two network architectures for solving this supervised learning problem—one, a minimalist feed-forward network, and the other, a fully-connected recurrent network, and compare the properties of the two architectures.

#### 3.3.1. Linear Feed-Forward Network

Define the following loss function for solving a linear classification problem

(52) $$\begin{array}{l} \underset{a_{i},b}{\min}L_{\text{linear}}=\underset{a_{i},b}{\min}|y-\overset{D}{\underset{i=1}{\sum }}a_{i}x_{i}-b| \end{array}$$

where *a*_*i*_ ∈ ℝ, *i* = 1, ..., *D*, are the feed-forward weights connecting the input features to the output, and *b* ∈ ℝ is the bias term. We can minimize this loss function by considering a feed-forward network only consisting of synaptic projections from input neuron pairs to the output neuron pair. Let the *i*-th input neuron pair be denoted by (*i*^+^, *i*^−^), *i* = 1, ..., *D*, and the output neuron pair be denoted by (*y*^+^, *y*^−^). The network also has a bias neuron pair denoted by (*b*^+^, *b*^−^) which receives a constant positive input equal to 1 for each data point. Feed-forward synaptic connections from the feature neuron pairs to the output neuron pair are then given by

(53) $$\begin{array}{l} \;Q_{yi}=a_{i},i=1,...,D,\\ Q_{yb}=b. \end{array}$$

Self-synaptic connections *Q*_*ii*_ are kept constant at 1 throughout training, while all remaining connections are set to zero. When we present a data point (***x**, y*) to the network, from (35) we have

(54) $$\begin{array}{l} \left(v_{i}^{+}-v_{i}^{-}\right)=x_{i},\;i=1,...,D,\text{ and} \end{array}$$

(55) $$\begin{array}{l} \left(v_{b}^{+}-v_{b}^{-}\right)=1. \end{array}$$

For the output neuron pair, we similarly have

(56) $$\begin{array}{l} v_{y}^{+}-v_{y}^{-}=y-\underset{j\neq y}{\sum }Q_{ij}\left(v_{j}^{+}-v_{j}^{-}\right)\\ \;=y-\overset{D}{\underset{i=1}{\sum }}a_{i}\left(v_{i}^{+}-v_{i}^{-}\right)-b\left(v_{b}^{+}-v_{b}^{-}\right)\\ \;=y-\overset{D}{\underset{i=1}{\sum }}a_{i}x_{i}-b. \end{array}$$

Then, minimizing the sum of mean firing rates for the output neuron pair gives us

(57) $$\begin{array}{l} \underset{Q_{yi},Q_{yb}}{\min}L_{ff}\left(Q_{yi},Q_{yb}\right)=\underset{Q_{yi},Q_{yb}}{\min}(\Psi_{y}^{+}+\Psi_{y}^{-})\\ \;=\underset{Q_{yi},Q_{yb}}{\min}|v_{y}^{+}-v_{y}^{-}|\\ \;=\underset{a_{i},b}{\min}|y-\overset{D}{\underset{i=1}{\sum }}a_{i}x_{i}-b|\\ \;=\underset{a_{i},b}{\min}L_{\text{linear}} \end{array}$$

**Figure 7:**
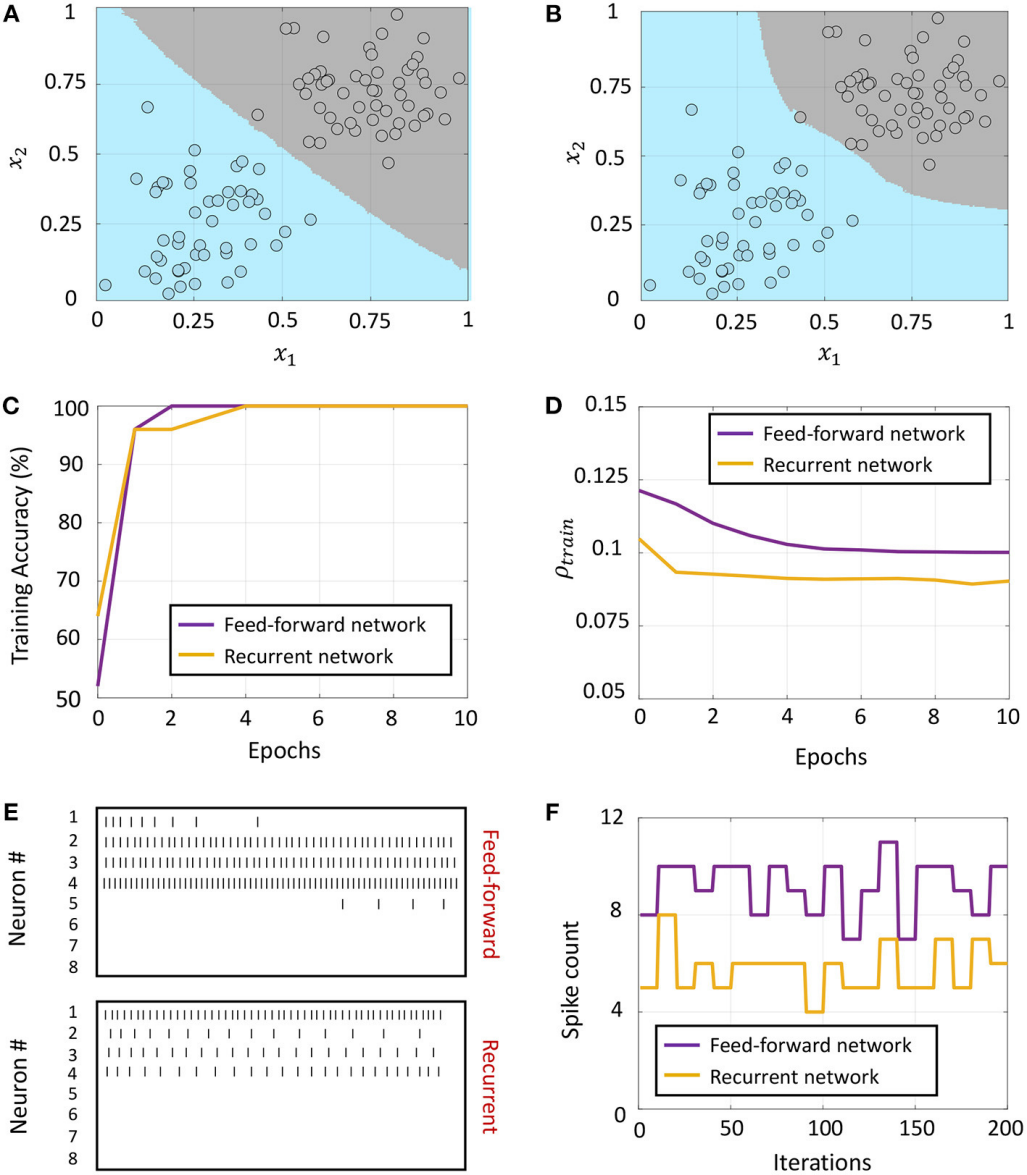
**(A,B)** Classification boundaries for a linearly separable, synthetic, two-dimensional binary dataset learned by a feed-forward network and a recurrently connected network respectively, with the two classes plotted in blue and gray. **(C)** Evolution of training accuracy with number of epochs for the two networks. **(D)** Evolution of the sparsity metric defined in (60) with number of epochs for the two networks. **(E,F)** Spike rasters and post-stimulus time histogram (PSTH) plots for one training data point after weight adaptation corresponding to the feed-forward and recurrent networks, respectively.

The linear classification framework with the feed-forward architecture is verified in Figure 7A for a synthetic two-dimensional binary dataset, where training data points belonging to the two classes are plotted as gray and blue circles. During inference, both possible labels are presented to the network along with each test data point, and the data point is assigned to the class that produces the least number of spikes across the output neurons, according to the inference procedure outlined in section 2.3.2. Figure 7A also shows the classification boundary produced by the GT network after training.

#### 3.3.2. Linear Recurrent Network

We also considered a fully-connected network architecture for linear classification, where the feature and bias neuron pairs were not only connected to the output pair, but to each other. There were also trainable recurrent connections from the output pair to the rest of the network. Then from (35) we can write

(58) $$\begin{array}{l} Qv=x^{\prime }, \end{array}$$

where $x^{\prime }={\left[y,x_{1},x_{2},...,x_{D},1\right]}^{T}$ is the augmented vector of inputs. We solve the following optimization problem for the recurrent network, which minimizes sum of firing rates for all neuron pairs across the network

(59) $$\begin{array}{l} \underset{Q}{\min}L_{fc}\left(Q\right)=\underset{Q}{\min}\overset{M}{\underset{i=1}{\sum }}\left(\Psi_{i}^{+}+\Psi_{i}^{-}\right). \end{array}$$

Weight adaptation in the fully-connected network ensures that (58) is satisfied with the minimum norm on the vector of membrane potentials, i.e., the lowest spiking activity across the network, as opposed to enforcing the sparsity constraint only on the output neuron pair in the previous case. The inference phase then proceeds as before by presenting each possible label to the network, and assigning the data point to the class that produces the least number of spikes across the network.

Figure 7B shows the classification boundary produced by the fully-connected network for the same two-dimensional binary dataset. We see that both networks are able to classify the dataset, although the final classification boundaries are slightly different. Figure 7C plots the training accuracy vs. number of epochs for the two networks, where each epoch refers to training on the entire dataset once. For comparing how the network-level spiking activity evolves with training for the two networks, we average the sparsity metric given in (50) over the training dataset

(60) $$\begin{array}{l} \rho_{\text{train}}=\frac{1}{2MK}\overset{K}{\underset{k=1}{\sum }}\overset{M}{\underset{i=1}{\sum }}\left(s_{b_{ki}}^{+}+s_{b_{ki}}^{-}\right), \end{array}$$

where $s_{b_{ki}}^{+}$ and $s_{b_{ki}}^{-}$ are the mean spike counts of the *i*-th ON-OFF pair when the *k*-th training data point is presented to the network along with the correct label. Figure 7D plots how this metric evolves with the number of training epochs for the two networks. Although the fully-connected network has a considerably higher number of non-zero synaptic connections, the final network firing activity after training has converged is much lower than the feed-forward network, indicating that it is able to separate the classes with a much lower network-level spiking activity across the entire training dataset. This is also evident from Figures 7E,F, which show the spike rasters and post-stimulus time histogram (PSTH) curves for one representative training data point corresponding to the feed-forward and fully-connected networks respectively, after the weights have converged for both networks. We see that the total spike count across the network is much lower for the fully-connected network.

### 3.4. Multi-Layer Spiking GTNN

We can now build up on the results from the previous sections to construct end-to-end spiking networks for solving more complex non-linearly separable classification problems. In this section, we show three different network architectures using one or more of the components described in the preceding sections.

#### 3.4.1. Network 1: Classification Based on Random Projections

**Figure 8:**
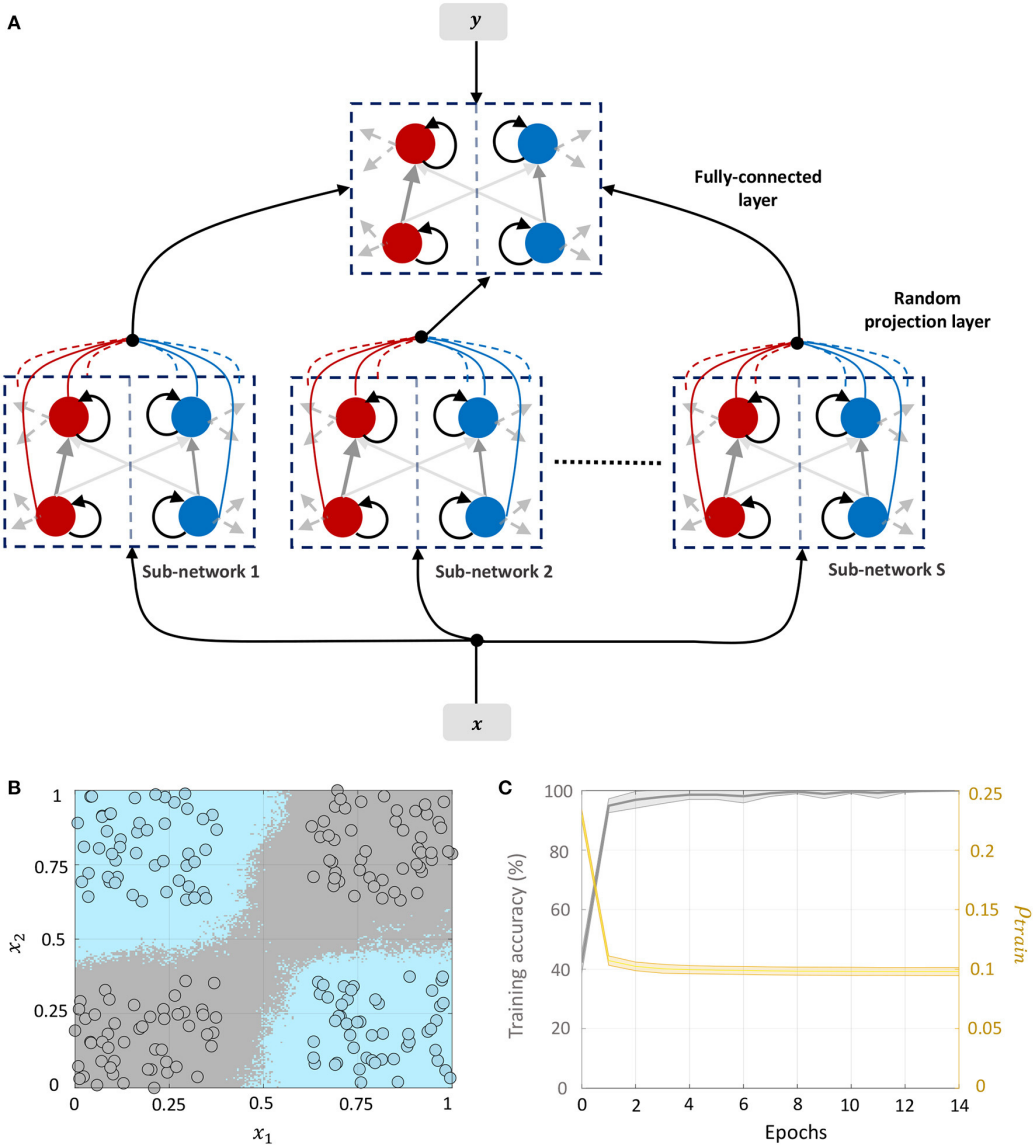
**(A)** Network architecture for non-linear classification based on random projections. **(B)** Classification boundary for a synthetic XOR dataset with a fully-connected network in the supervised layer, where the two classes are plotted in blue and gray. Contour plot corresponds to a majority vote over five trials with different initial conditions. **(C)** Plot of the average training accuracy and sparsity metric vs. number of training epochs over the five trials, with the shaded region denoting standard deviation about the mean.

This network architecture, shown in Figure 8A, consists of an unsupervised, random projection-based feature transformation layer followed by a supervised layer at the output. The random projection layer consists of *S* independent sub-networks, each with *D* differential neuron pairs, where *D* is the feature dimensionality of the training set. Let the transformation matrix for the *s*-th sub-network be denoted by ***Q***_*s*_ and its template vector be denoted by ***t***_*s*_, *s* = 1, ..., *S*. We consider a network configuration where each sub-network is densely connected, but there are no synaptic connections between the sub-networks. When the *k*-th training data point ***x***_*k*_ is presented to the network, we have from (51)

(61) $$\begin{array}{l} \overset{D}{\underset{i=1}{\sum }}\Psi_{ski}^{+}+\Psi_{ski}^{-}=-(\overset{D}{\underset{i=1}{\sum }}v_{ski}^{+}+v_{ski}^{-})\\ \;=||Q_{s}t_{s}-x_{k}{||}_{1}, \end{array}$$

where $\Psi_{ski}^{+}$ and $\Psi_{ski}^{-}$ are the mean values for the spike function of the *i*-th differential pair in the *s*-th sub-network, in response to the *k*-th data point, and $v_{ski}^{+}$ and $v_{ski}^{-}$ are the corresponding mean membrane potentials. We define the “centroid” for the *s*-th sub-network as

(62) $$\begin{array}{l} c_{s}=Q_{s}t_{s}. \end{array}$$

Thus, when a new data point ***x***_*k*_ is presented to the network, the sum of mean membrane potentials of the *s*-th sub-network essentially computes the *L*_1_ distance (with a negative sign) between its centroid ***c***_*s*_ and the data point. Note that no training takes place in this layer. The summed membrane potentials encoding the respective *L*_1_ distances for each sub-network serve as the new set of features for the linear, supervised layer at the top. Thus, for a network consisting of *S* sub-networks, the input to the supervised layer is an *S*-dimensional vector.

We demonstrate the random projection-based non-linear classification with the help of an XOR dataset in Figure 8B, which uses 50 sub-networks in the random projection layer and a fully-connected architecture in the supervised layer. The figure shows training data points belonging to the two classes as blue and gray circles, as well as the classification boundary produced by the GT network after five rounds of training with different initial conditions, as decided by a majority vote. Figure 8C plots the evolution of the mean and standard deviation of the training accuracy as well as the sparsity metric with each training epoch. Since training for this network architecture only takes place in the top layer, the sparsity gain is minimal.

#### 3.4.2. Network 2: Classification Based on Layer-Wise Training

**Figure 9:**
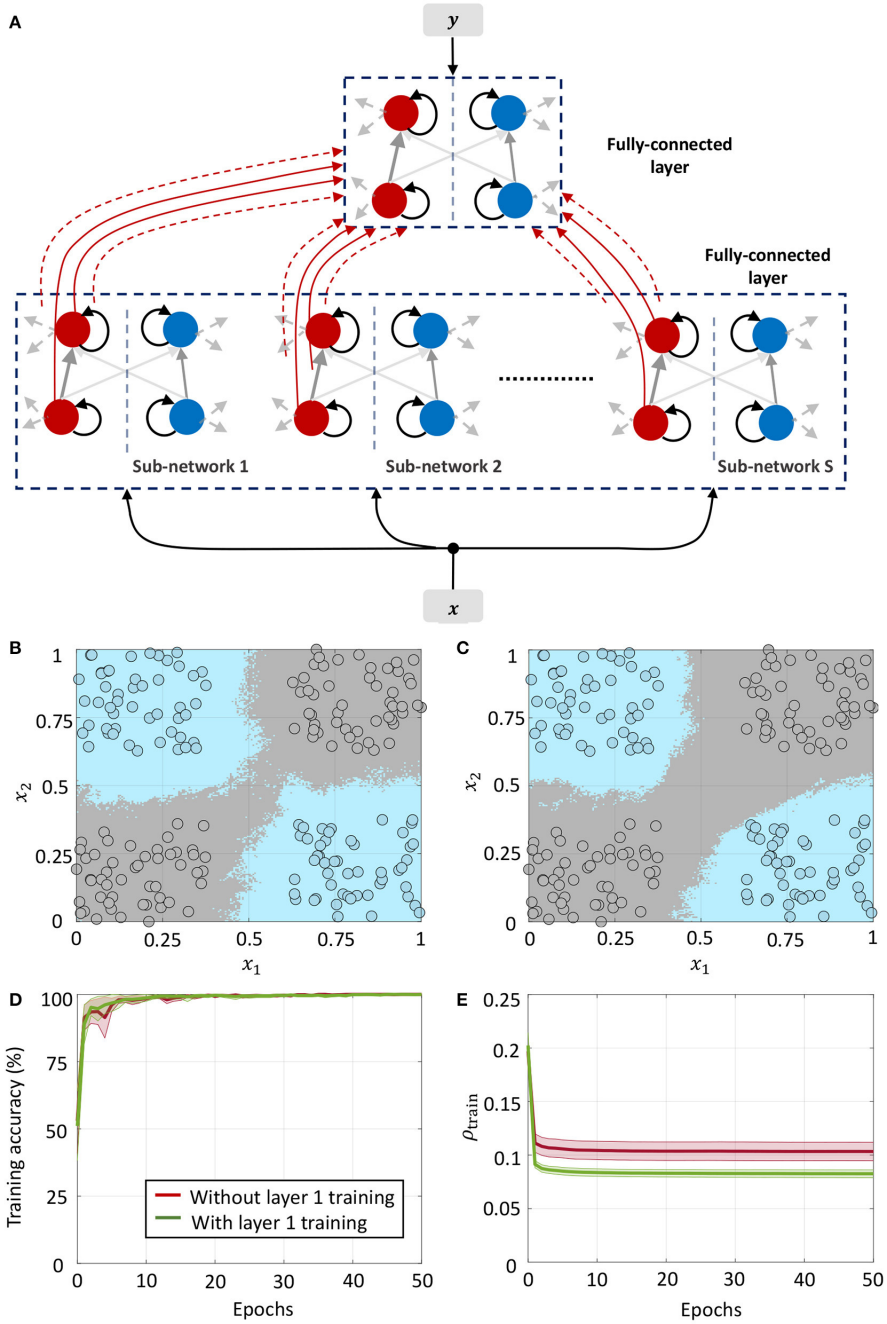
**(A)** Network architecture for non-linear classification based on layer-wise training. **(B,C)** Classification boundaries for the same XOR dataset without and with weight adaptation in layer 1, respectively. Contour plot corresponds to a majority vote over five trials with different initial conditions. **(D,E)** Mean training accuracy and mean sparsity metric vs. epochs for the two cases, estimated over five runs with random initializations.

The second network architecture, shown in Figure 9A, consists of two fully-connected differential networks stacked on top of each other, with connection matrices for the two layers denoted by ***Q***_1_ and ***Q***_2_, respectively. The first layer consists of *S* sub-networks as in the previous architecture, but with connections between them. For the first layer, we can rewrite (35) as follows

(63) $$\begin{array}{l} \;Q_{1}v_{1}=x_{1},\text{ or}\\ Q_{1}\left(v_{1}^{+}-v_{1}^{-}\right)=x_{1} \end{array}$$

where $v_{1}^{+},v_{1}^{-}\in {\mathbb{R}}^{M_{1}}$ are the vectors of mean membrane potentials for the ON and OFF parts of the differential network in layer 1, and $x_{1}={\left[x,x,...,x\right]}^{T}\in {\mathbb{R}}^{M_{1}}$ is the augmented input vector, *M*_1_ = *DS* being the total number of differential pairs in layer 1. Since for each neuron pair, only one of *v*^+^ and *v*^−^ could be non-zero, the mean membrane potentials for either half of the differential network encodes a non-linear function of the augmented input vector ***x***_1_, and can be used as inputs to the next layer for classification. We considered a fully-connected network in the second layer for linear classification. Figure 9B shows the classification boundary for the XOR dataset with this network architecture.

We can further train the first layer such that it satisfies (63) with much lower overall firing. Figure 9C shows the classification boundary for a network where synapses in both layers are adapted simultaneously at first, followed by adaptation only in the last layer. We see that the network learns a slightly different boundary, but is able to distinguish between the classes as before. This is also evident from Figure 9D, which plots the training accuracy vs. epochs for the two cases without and with adaptation in layer 1. Figure 9E plots the evolution of the sparsity metric evaluated on the training dataset for both cases. In the second case, the network learns to distinguish between the classes with a lower overall network-level spiking activity. Since both layers are trainable for this network, it is able to distinguish between the classes with a much lower network-level spiking activity for the dataset.

#### 3.4.3. Network 3: Including Target Information in Layer-Wise Training of Fully-Connected Layers

**Figure 10:**
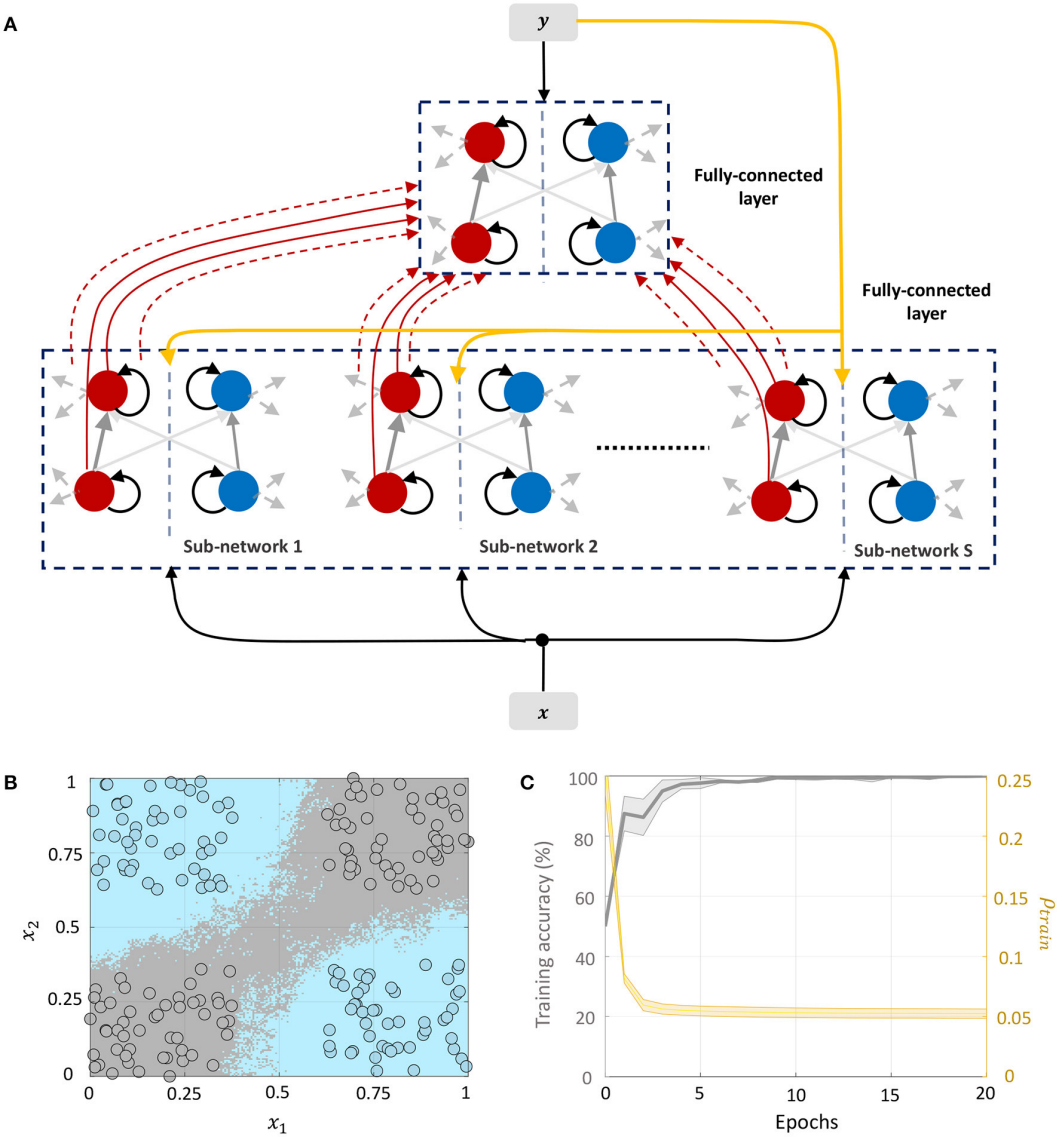
**(A)** Network architecture for non-linear classification based on layer-wise training with target information included. **(B)** Classification boundary for the XOR dataset where the contour plot corresponds to a majority vote over five trials with different initial conditions. **(C)** Plot of the average training accuracy and sparsity metric vs. number of training epochs over the five trials, with the shaded region denoting standard deviation about the mean.

We can drive the network to be sparser by including information about class labels in the layer-wise training of fully-connected layers. This allows the network to exploit any linear relationship between the elements of the feature and label vectors to further drive sparsity in the network. The corresponding network architecture is shown in Figure 10A. Each sub-network in layer 1 receives the external input ***x*** ∈ ℝ^*D*^ and the corresponding label vector ***y*** ∈ {−1, +1}^*C*^, where *C* is the number of classes. The top layer receives the *DS*-dimensional output vector corresponding to the membrane potentials from the positive part of layer 1 (which as in Network 2 encodes a non-linear function of its input) as well as the same label vector. Each layer in the network is trained with the correct class label. During inference, the network is presented with a new data point and the network-level spiking activity is recorded for each possible label vector. The data point is assigned to the class that produces the lowest firing across all layers of the network.

This architecture is similar to Direct Random Target Projection (Frenkel et al., 2021) which projects the one-hot encoded targets onto the hidden layers for training multi-layer networks. The notable difference, aside from the neuromorphic aspect, is that we use the input and target information in each layer to train the lateral connections within the layer, and not the feed-forward weights from the preceding layer. All connections between the layers remain fixed throughout the training process. Figure 10B shows the classification boundary for the XOR dataset with this network architecture, and Figure 10C shows the evolution of the training accuracy and sparsity metric for this problem with the number of training epochs.

### 3.5. Incremental, Few-Shot Learning on Machine Olfaction Dataset

A consequence of choosing the sparsest possible solution to the machine learning problem in the proposed framework is that it endows the network with an inherent regularizing effect, allowing it to generalize rapidly from a few examples. Alongside the sparsity-driven energy-efficiency, this enables the network to also be resource-efficient, making it particularly suitable for few-shot learning applications where there is a dearth of labeled data (Wang et al., 2020). In order to demonstrate few-shot learning with the proposed approach, we tested Networks 1–3 on the publicly available UCSD gas sensor drift dataset (Vergara et al., 2012; Rodriguez-Lujan et al., 2014). This dataset contains 13,910 measurements from an array of 16 metal-oxide gas sensors that were exposed to six different odors (ammonia, acetaldehyde, acetone, ethylene, ethanol, and toluene) at different concentrations. The measurements are distributed across 10 batches that were sampled over a period of 3 years, posing unique challenges for the dataset including sensor drift and widely varying ranges of odor concentration levels for each batch. Although the original dataset has eight features per chemosensor yielding a 128-dimensional feature vector for each measurement, the present work considers only one feature per chemosensor (the steady-state response level) resulting in a 16-dimensional feature vector, similar to other neuromorphic efforts on the dataset (Borthakur and Cleland, 2019).

#### 3.5.1. Previous Work on This Dataset

Previous efforts using classical machine learning approaches on this dataset include among others ensembling support vector machine classifiers trained at different points in time (Vergara et al., 2012), domain adaptation techniques using extreme learning machines (Zhang and Zhang, 2014; Ma et al., 2018) and transfer-sample based multi-task learning across domains using logistic regression (Yan et al., 2017). More recently, Borthakur and Cleland (2019) proposed a neuromorphic machine learning algorithm based on design principles from the mammalian olfactory circuit to extract relevant features, which were then assigned classes using a similarity metric based on Hamming distance.

#### 3.5.2. Our Approach

In order to mitigate challenges due to sensor drift, we followed the same reset learning approach as in Borthakur and Cleland (2019), re-training our network from scratch as each new batch became available using few-shot learning. However, the main objectives of the experiments in this section differ from previous work in the following ways:

- We demonstrate the proposed learning framework on a real-world dataset, where the network learns the optimal parameters for a supervised task by minimizing spiking activity across the network. We show that for all three architectures introduced previously, the network is able to optimize for both performance and sparsity. Moreover, we show that this is possible using a generic network that does not take into account the underlying physics of the problem.
- We showcase end-to-end, backpropagation-less spiking networks that implements feature extraction as well as classification within a single framework. Moreover, we demonstrate SNNs that can encode non-linear functions of layer-wise inputs using lateral connections within a layer, and present an approach to train these lateral connections.

**Figure 11:**
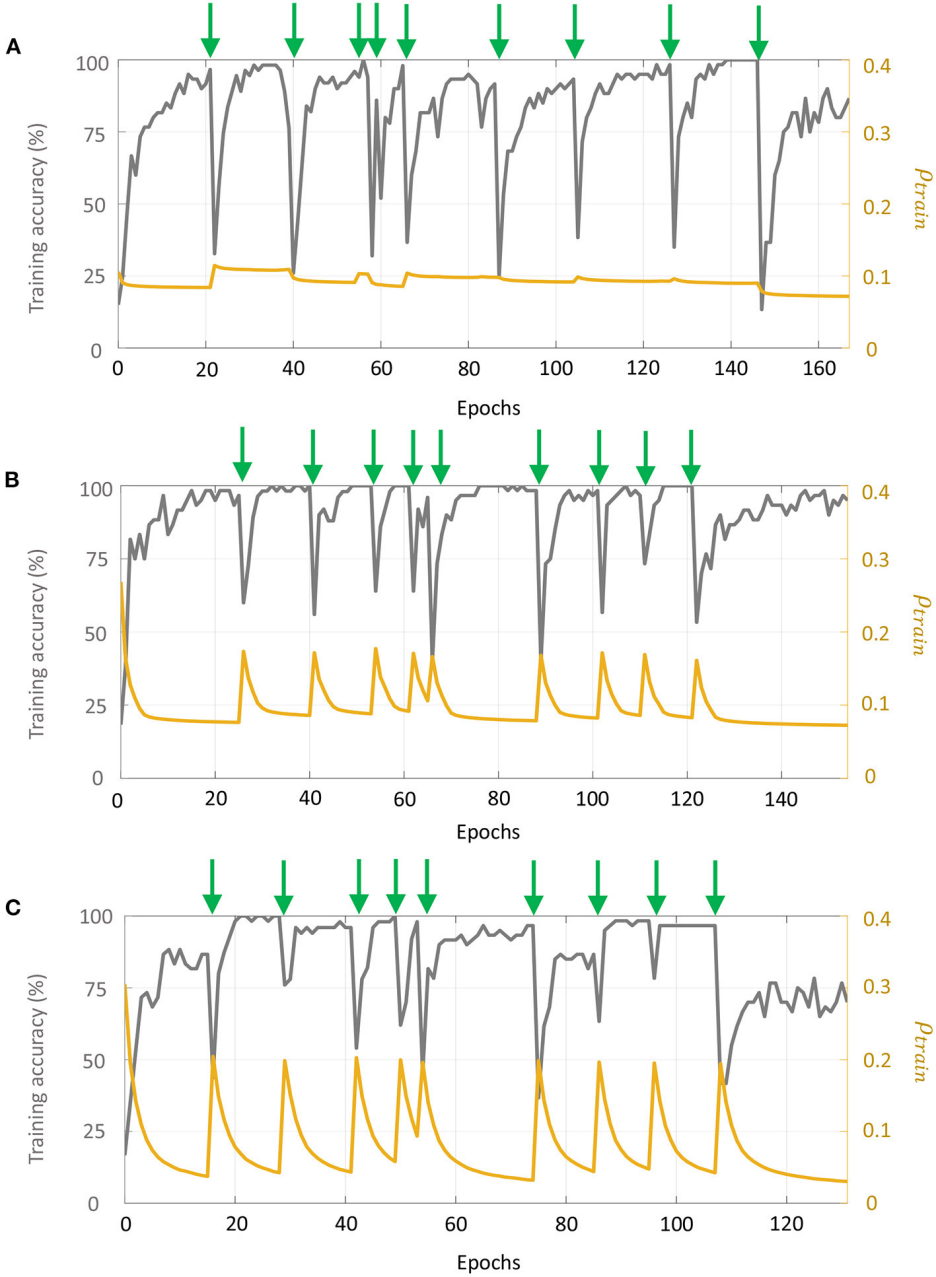
Evolution of the training accuracy and sparsity metric for **(A)** Network 1, **(B)** Network 2, and **(C)** Network 3 with each phase of re-training on the UCSD gas sensor drift dataset. Green arrows mark the beginning of each phase of re-training.

For each batch, we selected 10 measurements at random concentration levels for each odor as training data, and 10% of the measurements as validation data. The remaining data points were used as the test set. If a batch had fewer than 10 samples for a particular odor, we included all samples for that odor within the training set. Input features for the training data were scaled between [0, 1], and the same scaling parameters were used to transform the validation and test sets. For Network 1, we considered 50 sub-networks in the random projection layer, which produced a 50-dimensional input vector to the supervised layer. For Networks 2 and 3, the number of sub-networks in layer 1 was 20, generating a 320-dimensional input vector to layer 2 corresponding to the 16-dimensional input vector to layer 1. Moreover, for the first layer in Networks 2–3, we considered a connection probability of 0.5, randomly setting around half of the synaptic connections to 0. Figures 11A–C plot the evolution of training accuracy and sparsity metric (evaluated on the training set) with number of epochs for each phase of re-training for Networks 1–3, respectively. Green arrows in each plot mark the onset of a re-training phase, i.e., a new batch. We see that with the arrival of new training data in each batch and for each network, the training error as well as the average spiking activity across the network increases, and subsequently decline with re-training.

**Figure 12:**
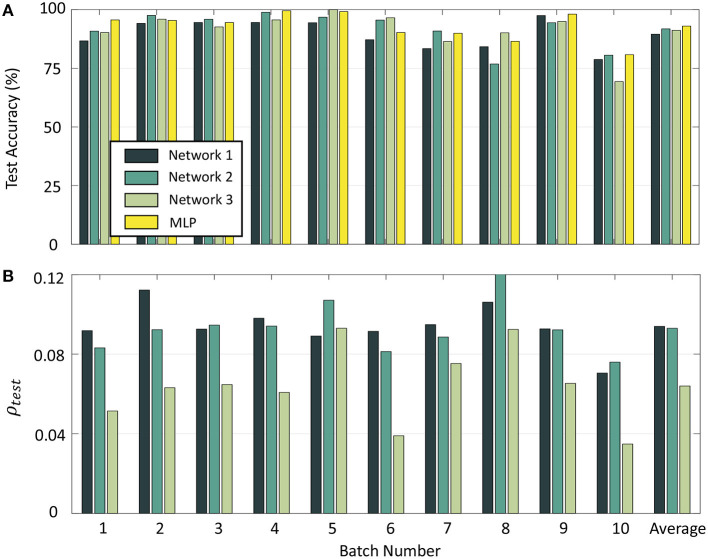
**(A)** Test accuracy on Batches 1–10 with Networks 1–3 and a Multi-layer Perceptron. **(B)** Sparsity metrics for Batches 1–10 with the three GTNN architectures.

To compare the performance of our network with standard backpropagation, we trained a multi-layer perceptron (MLP) with 16 inputs and 100 hidden units for the odor classification problem with a constant learning rate of 0.01 and using the same validation set as before. The number of hidden neurons as well as learning rate were selected through hyper-parameter tuning using only the validation data from Batch 1. Table 1 gives the number of measurements for each batch, as well as final test accuracies and sparsity metrics (evaluated on the test sets) for each batch for Networks 1–3 with 10-shot learning, as well as the final test accuracies for each batch with the multi-layer perceptron. Figure 12A shows the batch-wise test accuracies for the three GTNN architectures and the MLP, and Figure 12B shows the sparsity metrics on test data for the GTNN architectures. We see that the proposed networks produce classification performance comparable with classical backpropagation-based models, while driving sparsity within the network. The sparsity metrics are highest for Network 1, where synaptic adaptation takes place only in the top layer. Network 2 has the flexibility of training both layers, leading to a decline in the values of the sparsity metric for most batches. In Network 3, synaptic adaptation in both layers coupled with the inclusion of target information drives the sparsity values to less than half of Networks 1–2.

**Table 1:** Batch-wise information, final test accuracies, and sparsity metrics evaluated on test data for the UCSD gas sensor drift dataset with Networks 1–3 and with a Multi-layer Perceptron network.

**Batch**		**1**	**2**	**3**	**4**	**5**	**6**	**7**	**8**	**9**	**10**
Data points		445	1,244	1,586	161	197	2,300	3,613	294	470	3,600
N/w 1	Acc. (%)	86.72	94.17	94.55	94.56	94.44	87.20	83.42	84.24	97.50	78.77
	ρ_*test*_	0.0918	0.1122	0.0926	0.0981	0.0891	0.0915	0.0949	0.1061	0.0927	0.0705
N/w 2	Acc. (%)	90.86	97.55	95.93	98.91	96.83	95.57	90.90	76.85	94.44	80.60
	ρ_*test*_	0.0832	0.0923	0.0946	0.0941	0.1071	0.0813	0.0886	0.1207	0.0922	0.0760
N/w 3	Acc. (%)	90.26	95.95	92.65	95.65	100.00	96.56	86.42	90.14	95.00	69.34
	ρ_*test*_	0.0514	0.0631	0.0647	0.0608	0.0930	0.0390	0.0753	0.0924	0.0653	0.0348
MLP	Acc. (%)	95.63	95.42	94.53	99.56	99.20	90.27	89.96	86.50	98.11	80.81

**Figure 13:**
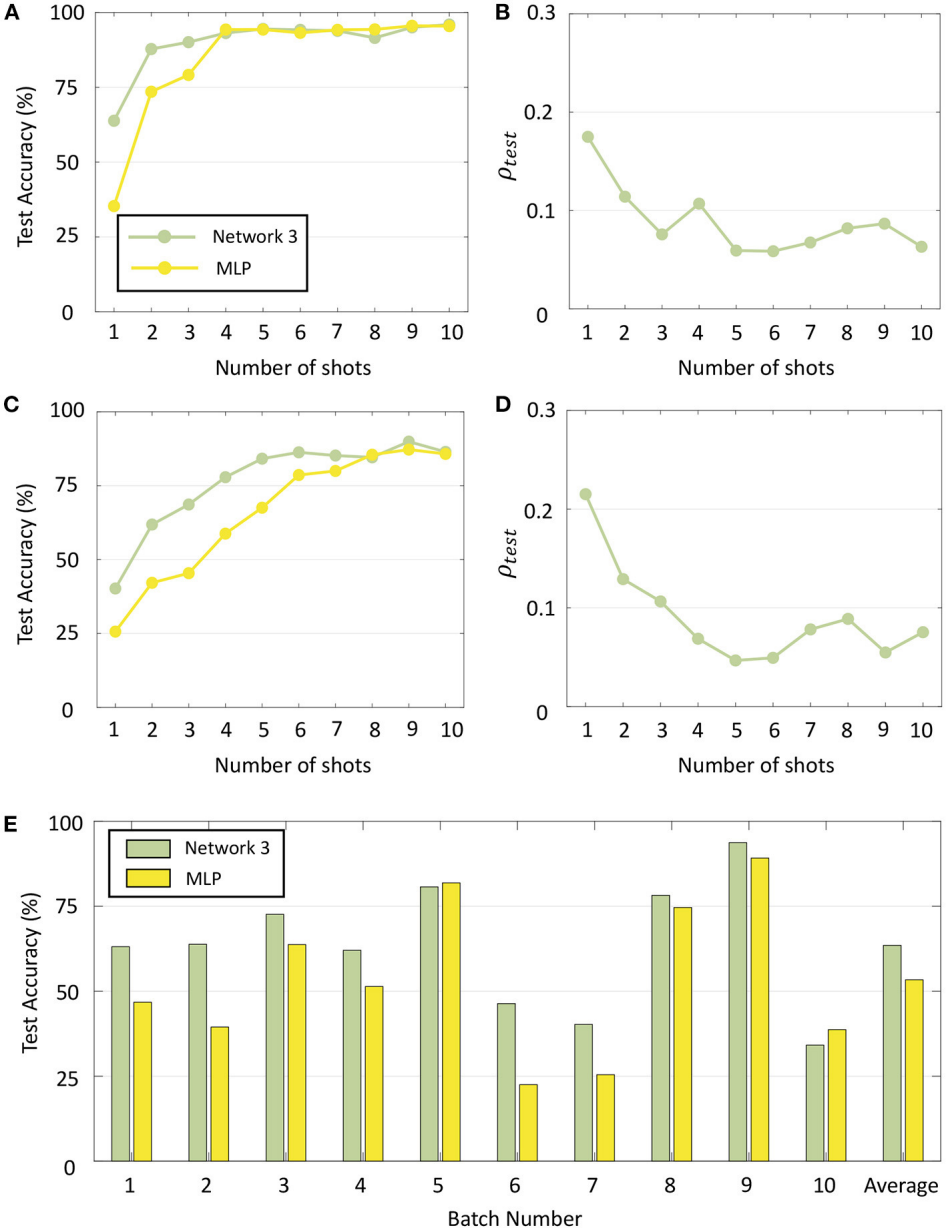
**(A)** Test accuracy for Batch 2 with Network 3 and a standard MLP when the number of shots is varied from 1 to 10. **(B)** Sparsity metrics corresponding to **(A)**. **(C,D)** Same plots for Batch 7. **(E)** Test accuracy for Batches 1–10 with Network 3 and MLP with one-shot learning.

When the number of shots, i.e., the number of training data points per class for each phase of re-training is further reduced, the classification performance of GTNN declines more gracefully than standard learning algorithms when no additional regularizing effect or hyper-parameter tuning was done. This is demonstrated in Figures 13A,C for two representative batches (Batch 2 and Batch 7, respectively) where the test accuracy with Network 3 as well as with MLP is plotted by varying the number of shots from 1 to 10. Importantly, no hyper-parameters were changed from the previous experiments in order to evaluate recognition performance when no such tuning would be possible under different training scenarios. It is seen that although MLP yields similar classification performance as GTNN for a larger number of shots, GTNN has a consistently higher recognition performance for fewer training data points per class. Figures 13B,D plot the corresponding test sparsity metrics, which show that the network becomes sparser as the network is trained on more and more data points. Figure 13E plots the test accuracy of Batches 1–10 with the same two networks for one-shot learning. For most of the batches, GTNN performs significantly better.

### 3.6. Incremental Learning on Other Benchmark Datasets

We evaluated the performance of Networks 1–3 on two other benchmark datasets, “Wisconsin Breast Cancer” (Wolberg and Mangasarian, 1990) and “Diabetes” from the UCI Machine Learning Repository (Dua and Graff, 2019). Seventy percent of the data points were randomly selected for training, and another 20% for validation. Table 2 summarizes the test accuracies and sparsity metrics (evaluated on test data) using incremental learning. Each dataset is labeled with attributes (N, D, M), which refer to the size of dataset, number of dimensions and number of classes, respectively.

**Table 2:** Final test accuracies and sparsity metrics evaluated on test data for different datasets.

**Dataset**	**Network 1**		**Network 2**		**Network 3**	
	**Test accuracy (%)**	**ρ_*test*_**	**Test accuracy (%)**	**ρ_*test*_**	**Test accuracy (%)**	**ρ_*test*_**
Breast cancer (699, 9, 2)	98.53	0.0653	95.59	0.0647	97.06	0.0514
Diabetes (768, 8, 2)	73.68	0.0723	73.68	0.0965	73.68	0.0191
AReM task 1 (30, 6, 480, 2)	–	–	–	–	100.00	0.0474
AReM task 2 (37, 6, 480, 3)	–	–	–	–	100.00	0.0370
AReM task 3 (36, 6, 480, 3)	–	–	–	–	90.00	0.0347

GTNN can also be used as a recurrent network for capturing dynamic information time-series data by letting the input vector change slowly over the training duration, while the output labels are held constant. This is similar to liquid state machines (Maass et al., 2002), with the difference that the recurrent connections are adapted here instead of the feed-forward weights connecting the recurrent neurons to the output units, as usually done in reservoir computing-based approaches. To demonstrate this, we ran experiments with Network 3 using the Activity Recognition system based on Multisensor data fusion (AReM) dataset, which contains time-series data generated by a Wireless Sensor Network (WSN) worn by an user performing seven different activities (Palumbo et al., 2016). For each data point, there are six different sequences containing information about the Received Signal Strength (RSS) values coming from the WSN, where each sequence consists of 480 time-steps corresponding to 120 s. The dataset contains 88 such sequences with a total of 42,240 instances. Specifically, we consider three tasks proposed in (Palumbo et al., 2016), each focusing on discriminating between smaller sets of similar movements: Task 1 involves Cycling and Standing, Task 2 involves Bending1, Standing and Walking; and Task 3 involves Bending2, Lying and Sitting. We used 18, 21, and 20 sequences, respectively for training Network 3 on Tasks 1–3, and another 4, 6, and 6 sequences, respectively for validation. Each sub-network in Network 3 comprised six differential neuron pairs to process the sequences corresponding to each activity. Results on the AReM dataset are also given in Table 2, where dataset labels (N, D, L, M) refer to the size of dataset, number of sequences per data point, sequence length, and number of classes, respectively.

## 4. Discussion

In this paper we presented a learning framework for the Growth transform neural network that is able to learn optimal parameters for a given task while simultaneously minimizing spiking activity across the network. We demonstrated how the same framework could be used in different network configurations and settings to solve a range of unsupervised and supervised machine learning tasks, and presented results for benchmark datasets. We also showed how the sparsity-driven learning endows GT network with an inherent regularizing effect, enabling it to generalize rapidly from very few training examples per class. Since only the lateral connections within each layer are trained, the network structures considered in this paper are inherently scalable where each layer can be independently and simultaneously trained, eradicating the need for propagating error gradients throughout the network. The sparsity-driven learning also ensures that the neurons remain within their dynamic ranges and away from saturation, facilitating the training of larger networks. Moreover, sparsity optimization not only reduces the number of active neurons, but also the spiking rates of the active neurons, and as such could be used to relax the communication bandwidth and improve network scalability.

**Figure 14:**
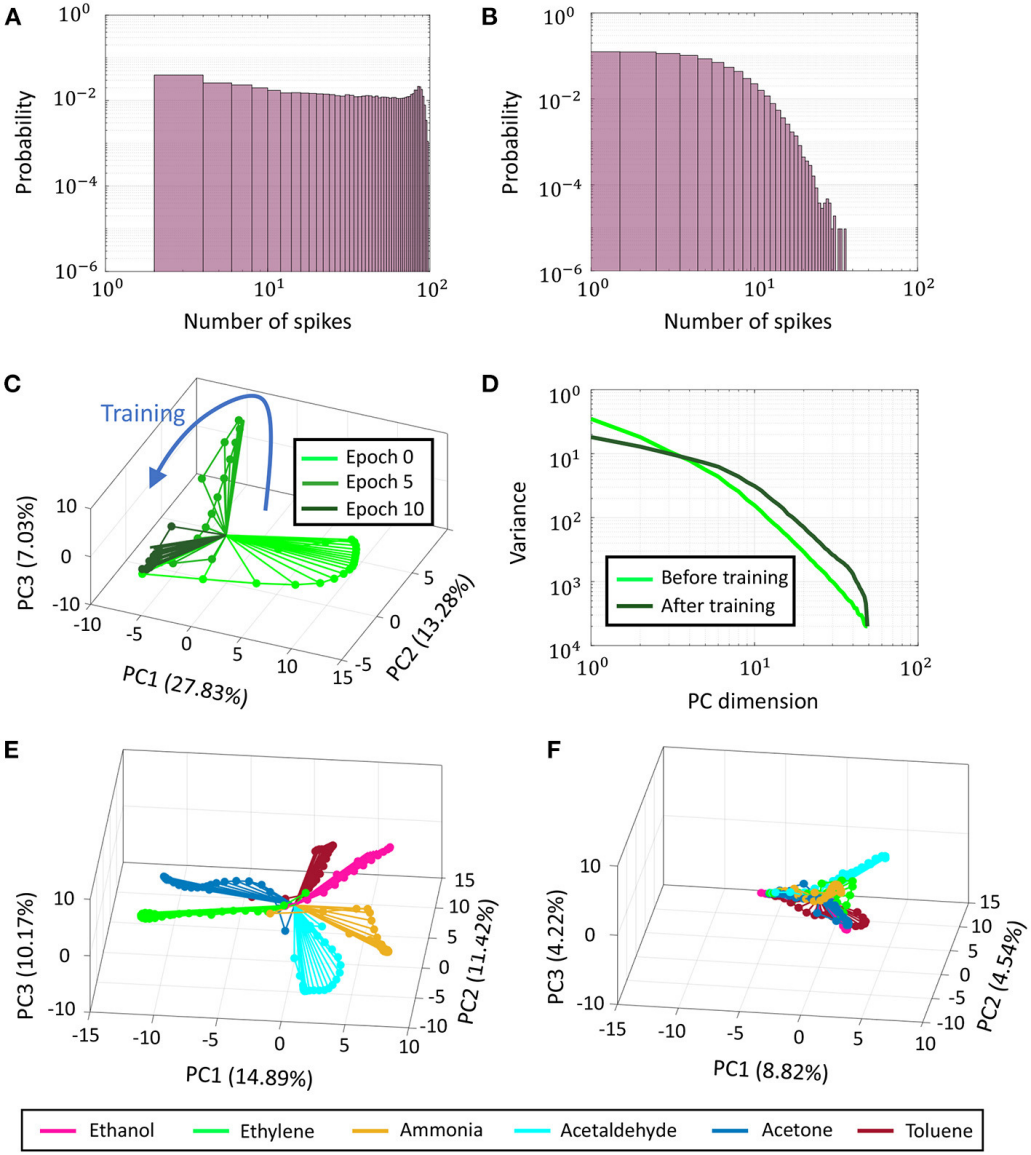
**(A)** Probability distribution of spike counts of individual neurons across Network 3 for a training data point before training. **(B)** Same plot post-training. **(C)** Evolution of PCA trajectories from normalized binned spike counts corresponding to a single training data point as training progresses in Network 3. **(D)** Plot of variance vs. principal component dimension averaged over 6 training data points belonging to six different odors, before and after training. **(E,F)** PCA trajectories before and after training for the same data points as in **(D)**.

A deeper analysis of the network and the synaptic dynamics reveals several parallels and analogies with dynamics and statistics observed in biological neural networks. Figure 14 shows how the population activity evolves in the GT network with synaptic adaptation using the proposed learning framework. Figures 14A,B plot the probability histograms of spike counts elicited by individual neurons in response to the same set of stimuli before and after training in log-log scale. There is a distinct shift in the range of spike counts from higher to lower values observed before and training, as expected in sparsity-driven learning.

Figures 14C,E,F present how network trajectories evolve as training progresses. Stimulus encoding by a population of neurons is often represented in neuroscientific literature by a trajectory in high-dimensional space, where each dimension is given by the time-binned spiking activity of a single neuron. This time-varying high-dimensional representation can be projected into two or three critical dimensions using dimensionality reduction techniques like Principal Component Analysis (PCA) or Linear Discriminant Analysis (LDA) to uncover valuable insights about the unfolding of population activity in response to a stimulus (Friedrich and Laurent, 2001; Stopfer et al., 2003). In Figure 14C, we plot the evolution of PCA trajectories of normalized binned spike counts across Network 3 with training corresponding to a training data point belonging to odor 1 (Ethanol). The percentages in the axes labels indicate the percentage of variance explained by the corresponding principal component. For the sake of clarity, we only show trajectories at three instances of time—before training, halfway into training and after training. With synaptic adaptation, the PCA trajectories are seen to shrink and converge faster to the steady-state, indicating the network-level spiking has reduced. This is also evident from Figures 14E,F, which plot network trajectories for training data points belonging to six different classes (odors) before and after training, respectively on the same subspace. Although the trajectories belonging to different classes are elaborate and clearly distinguishable before training, they are seen to shrink and become indistinguishable after training. Moreover, the net percentage of variance explained by the first 3 principal components decreases from ≈37% to only ≈18%. Together, these indicate that stimulus representation becomes less compressible with training, relying on fewer spikes across a larger population of neurons to achieve the optimal encoding. Figure 14D shows the decay in the eigenspectra corresponding to the PCA plots shown in Figures 14E,F. Both the spectra, pre-training and post-training, exhibit a power-law decay, a property that has been observed in evoked population activity in biological neurons (Stringer et al., 2019). However, as shown in Figure 14D, the eigenspectrum post-training reveals that the network encodes its activity with a code that is higher-dimensional than pre-training. The tails of the spectrum, though, decays faster indicating that the trained GTNN network efficiently utilizes the high-dimensional code to represent neural activity.

### 4.1. Implications for Neuromorphic Hardware

The GT neuron and network model, along with the proposed learning framework, has unique implications for designing energy-efficient neuromorphic hardware, some of which are outlined below:

- Typically in neuromorphic hardware, transmission of spike information between different parts of the network consumes most of the active power (Sorbaro et al., 2020). This paper presents a learning paradigm that can drive the network to converge to an optimal solution for a learning task while minimizing firing rates across the network, thereby ensuring performance and energy optimality at the same time.
- Unlike most spiking neural networks which adapt feed-forward weights connecting one layer of the network to the next, the proposed framework presents an algorithm for weight adaptation between the neurons in each layer, while keeping inter-layer connections fixed. This could significantly simplify hardware design as the network size scales up, where neurons in one layer could be implemented locally on a single chip, reducing the need for transmitting weight update information between chips. Moreover, unlike backpropagation, this algorithm can support simultaneous and independent weight updates for each layer, eradicating reliance on global information. This could enable faster training with less memory access requirements.

### 4.2. Relation With Balanced Spiking Networks

The balance between excitation and inhibition has been widely proposed to justify the temporally irregular nature of firing in cortical networks frequently observed in experimental recordings. This balance ensures that the net synaptic input to a neuron are neither overwhelmingly depolarizing nor hyper-polarizing, dynamically adjusting themselves such that the membrane potentials always lie close to the firing thresholds, primed to respond rapidly to changes in the input (Van Vreeswijk and Sompolinsky, 1996).

The differential network architecture considered in our paper is similar in concept, maintaining a tight balance between the net excitation and inhibition across each differential pair. We show how designing the network this way always satisfies a linear relationship between the mean membrane potentials and the external inputs. Moreover, we propose a learning framework that adapts the weights of the differential network such that membrane potentials of both halves of the differential pairs are driven close to their spike thresholds, minimizing the network-level spiking activity. By appropriately designing the network, it was shown how this property could be exploited to simultaneously minimize a training error to solve machine learning tasks.

## Data Availability Statement

Publicly available datasets were analyzed in this study. All datasets can be found at http://archive.ics.uci.edu/ml. The MATLAB scripts implementing GTNN learning and inference can be found at https://github.com/aimlab-wustl/GTNN-Learning.

## Author Contributions

AG and SC contributed to the conception, design of the study, and wrote the first draft of the manuscript. AG conducted the simulations. All authors contributed to the manuscript revision, read, and approved the submitted version.

## Conflict of Interest

The authors declare that the research was conducted in the absence of any commercial or financial relationships that could be construed as a potential conflict of interest.

## Publisher's Note

All claims expressed in this article are solely those of the authors and do not necessarily represent those of their affiliated organizations, or those of the publisher, the editors and the reviewers. Any product that may be evaluated in this article, or claim that may be made by its manufacturer, is not guaranteed or endorsed by the publisher.
